# Enhancing structural control in covalent organic frameworks through steric interaction-driven linker design†

**DOI:** 10.1039/d4sc03461a

**Published:** 2024-08-13

**Authors:** Alena Winter, Farzad Hamdi, Andreas Eichhöfer, Kay Saalwächter, Panagiotis L. Kastritis, Frederik Haase

**Affiliations:** a Institute of Chemistry, Faculty of Natural Sciences II, Martin-Luther-Universität Halle-Wittenberg Halle/Saale Germany frederik.haase@chemie.uni-halle.de; b Department of Integrative Structural Biochemistry, Institute of Biochemistry and Biotechnology, Martin Luther University Halle-Wittenberg Halle/Saale Germany; c Interdisciplinary Research Center HALOmem, Charles Tanford Protein Center & Biozentrum, Martin Luther University Halle-Wittenberg Halle/Saale Germany; d Institute for Nanotechnology (INT), Karlsruhe Institute of Technology (KIT) Eggenstein-Leopoldshafen Germany; e Institute of Physics, Martin-Luther-Universität Halle-Wittenberg Halle/Saale Germany; f Institute of Chemical Biology, National Hellenic Research Foundation Athens Greece; g Institute of Functional Interfaces (IFG), Karlsruhe Institute of Technology (KIT) Eggenstein-Leopoldshafen Germany

## Abstract

Covalent Organic Frameworks (COFs) exhibiting kagome (*kgm*) structures are promising crystalline porous materials with two distinct pores. However, there are no reliable synthetic methods to exclusively target the *kgm* over the polymorphic square-lattice (*sql*) structure. To address this, we introduce a linker design strategy featuring bulky functional groups, which through steric interactions can hinder the *sql* net formation, thereby leading to a *kgm* structure. By rigid attachment of the methyl benzoate groups to a tetradentate COF linker, steric interactions with neighbouring linkers depending on the pore size become possible. The steric interaction was tuned by varying the complementary bidentate linear linker lengths, where the shorter phenylenediamine linker leads to steric hindrance and the formation of the *kgm* lattice, while with the longer benzidine linker, steric interaction is reduced leading to the *sql* lattice. Thus, control over the net can be exerted through steric interaction strengths. Additionally, structural analysis revealed the formation of the *kgm* COF with an unusual ABC stacking, leading to pearl string type pores instead of two distinct pore sizes. This COF system shows that steric interaction-driven design enhances control over COF structures, expanding the design toolbox, but also provides valuable insights into network formation and polymorphism.

## Abstract 2

Using a linker with bulky functional groups encourages kagome formation, while blocking square-lattice network formation in Covalent Organic Frameworks (COFs).

## Graphical abstract

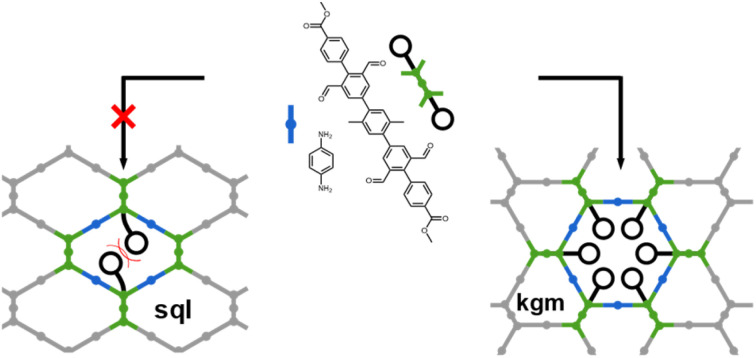

## Introduction

The appeal of covalent organic frameworks (COFs), and reticular materials in general, stems from their predictable structures where the geometry of the building blocks determines the resulting net. However, with certain building block geometries, the outcome of a synthesis cannot always be predicted due to polymorphism. In three dimensional COFs,^1–3^ a significant challenge arises from the vast array of potential structures (and nets) that can emerge from a single type of building block. In two-dimensional COFs, net polymorphism is mostly known from the competition between kagome (*kgm*) and square lattice (*sql*) when using planar four connected (4-c) linkers. Notable further examples of (pseudo-) polymorphism are frustrated COFs based on three connected (3-c) with a 4-c linker leading to sub-stochiometric 2D COFs.^4–6^

**Fig. 1:**
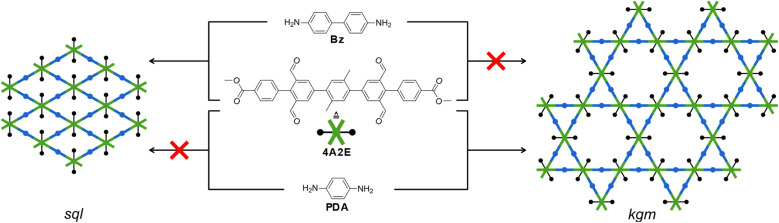
Schematic depiction of the possible formed nets (*sql*, left and *kgm*, right; blue: 2-connected (2-c) and green: 4-c).

The competition of *kgm vs. sql* nets occur as 4-c building blocks, with angles of 60° and 120° between the functional groups, can construct both nets without distorting the building blocks (Fig. 1).^7,8^ The *kgm* topology has generated much interest because it contains two differently-sized pores in one structure and can lead to interesting optical and electronic properties.^9^ Targeting just one of these nets remains an unresolved challenge in COFs. Efforts have been undertaken to control the topology using different linkers. For example, by controlling the planarity of the building block^10^ or with a hydrogen-bonding stabilized imine.^11^ In many examples, tetraphenylethylene-based building blocks prefer to form the *kgm* structure,^7,8,12,13^ while pyrene-based linkers prefer to form *sql* nets.^14–18^ This suggests that the 4-c linkers govern the topology of the formed COFs, yet it is often not clear, why a system prefers either *kgm* or *sql* structures. In some systems this predetermination of the topology can be overcome with different synthesis conditions.^1,19–21^ Theoretic simulations have predicted that depending on the concentration and interaction of the solvent with the monomer, the nucleation of either *kgm* or *sql* should be possible.^22^

A much more well-understood type of polymorphism in 2D COFs is the so-called stacking modes, which describe the offset and sequence of layers. For structure elucidation, the stacking is often considered without disorder.^23^ Stacking modes can be differentiated by the offset between different layers. When the layers have no offset vector (0 Å offset), the stacking mode is referred to as eclipsed (*i.e.* AA stacking sequence). However, this idealized stacking is often assumed due to averaging of random offset stacking^24^ and is only rarely observed in reality.^13^ Alternately, uniform slipping of the layers in one direction (1–2 Å offset) can be observed.^25^ If the backbone of the COF is positioned over the centre of the pore in the layer below, the stacking is called staggered or AB/ABC stacked.^26^ Eclipsed and slipped stacking has important implications for the electronic properties of the material,^26^ which can be experimentally controlled by the addition of side chains,^27^ using donor–acceptor strategies,^25,28,29^ or introducing additional interactions between the layers.^30^ Staggered stacking is a strategy for generating small pore COFs, which is an important feature for porous materials in gas separation.^31^ Yet, only very few examples of staggered hexagonal honeycomb (*hcb*) COFs have been reported where the stacking can be controlled. A few examples realize this control through tuning steric interactions between the layers,^32^ addition of a modulator^33^ or synthesis conditions.^34^ Despite this apparent control over the stacking, the ABC stacking mode in other nets is exceedingly rare. For example, to the best of our knowledge, it has only been reported once for the kagome dual (*kgd*)^35^ and *kgm*^36^ topology.

In this study, we investigated how steric interactions through the pores of 2D COFs affect their nets. We achieved the through–pore interactions by rigid attachment of a long and bulky methoxycarbonyl-terminated phenyl group to a tetraaldehyde linker (4A2E). Using this modified tetraaldehyde, we synthesized COFs with different linear diamine linkers. We observed a *kgm* structure with the short *p*-phenylenediamine (PDA) and a *sql* structure with the longer benzidine (*p*-diaminodiphenyl, BZ). The structural characterization by powder X-ray diffraction (PXRD) and transmission electron microscopy (TEM) confirmed the selectivity for *sql* and *kgm*. Additionally, we found evidence for the rare ABC stacking in the *kgm* COF. Force field simulations provided evidence for strong steric crowding in the rhombic pore of a hypothetical *sql*4A2E-PDA-COF, based on the *p*-phenylenediamine. This steric crowding is relieved when forming the hexagonal pores in the *kgm* net. The longer benzidine linker increases the intrapore distance and decreases the steric demand, thus allowing the formation of a *sql* net. Our linker design enables sterically demanding functional groups to control the net. This design principle expands the COF synthesis toolbox for inducing the *kgm* net more reliably.

## Results

We designed a tetraaldehyde linker (Fig. 1 centre) based on a terphenyl core with two aldehydes attached to each outer phenyl ring. The aldehydes are attached in 3 and 5 positions to form a 60° and a 120° bite angle between them, which allows the formation of both *kgm* and *sql* networks. The linker core was functionalized in the 60° bay with methyl groups to increase the solubility and the 120° bay was functionalized with bulky methyl benzoate moieties. The linker 4A2E was synthesized in a three-step synthesis starting from 2-bromoisopthalaldehyde (for details see ESI Method section†).

### Synthesis

We employed the 4A2E linker for the synthesis of imine-based COFs using either phenylenediamine or benzidine. The synthesis was performed as a solvothermal approach in a mixture of 1,4-dioxane and mesitylene, with trifluoroacetic acid (TFA) serving as the catalyst. After the reaction, the precipitated COFs were filtered off and washed thoroughly with methanol, followed by Soxhlet extraction with methanol and activation by supercritical CO_2_. After extensive screening of the solvent ratios and acid concentrations, we obtained two crystalline COFs 4A2E-PDA-COF and 4A2E-BZ-COF, using PDA and BZ as linear diamines, respectively.

### Structural characterization

**Fig. 2:**
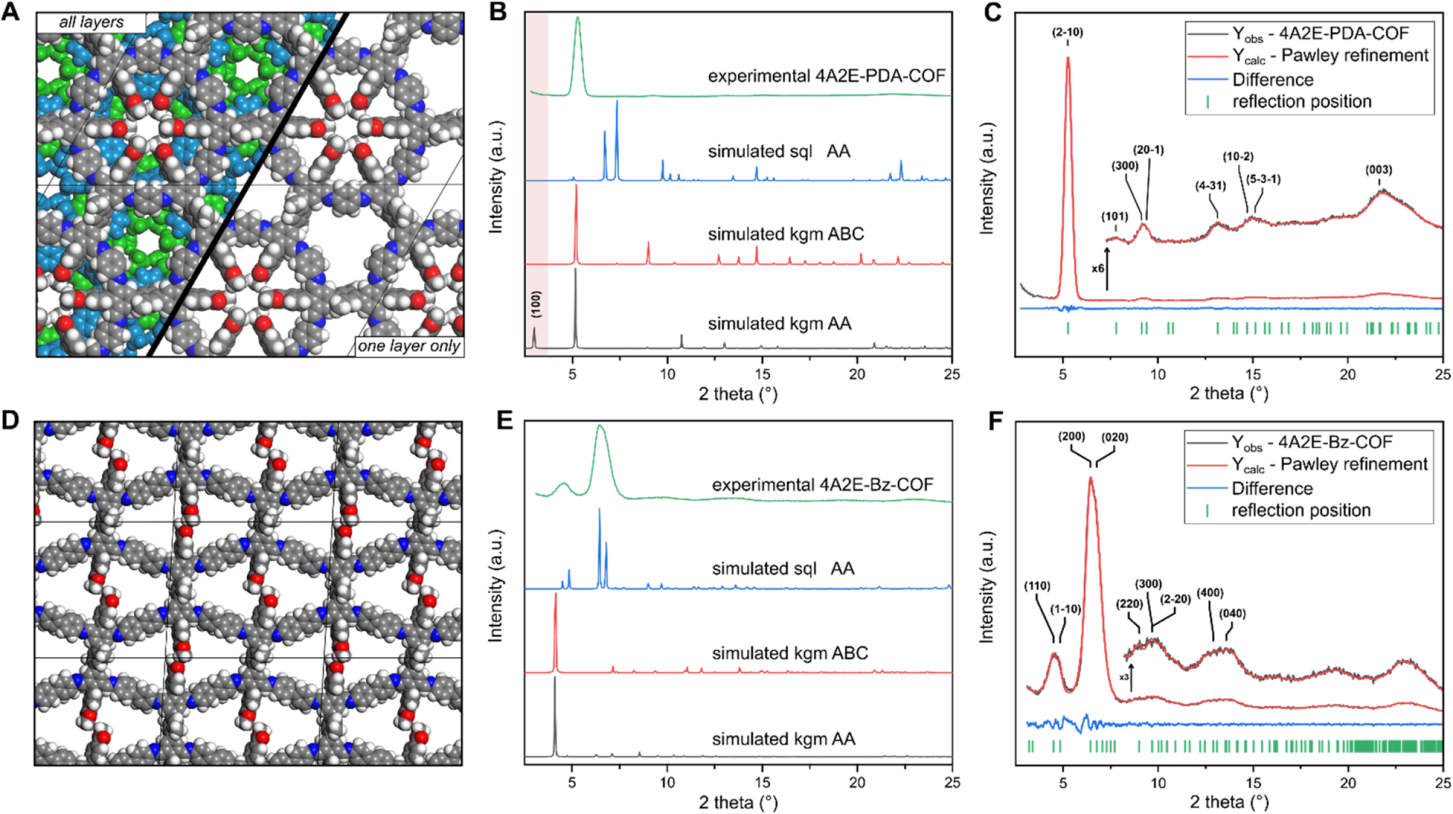
Simulated structures of 4A2E-PDA-COF (A) and 4A2E-BZ-COF (D), experimental PXRD compared to the simulated patterns (B and E) and Pawley refinement of the PXRD patterns (C and F).

The crystallinity of both 4A2E-PDA-COF and 4A2E-BZ-COF, was confirmed by PXRD (Fig. 2B and E). The PDA based COF showed sharper reflections and reflections visible up to higher angles indicating a higher crystallinity than the BZ based COF. As the linker geometry of 4A2E combined with a linear diamine leads to two possible 2D nets, *sql* and *kgm*, we constructed structural models of both nets in different stacking modes in Materials Studio. Based on these models, we simulated PXRD patterns (Fig. 2B and E).

#### 4A2E-BZ-COF

**Fig. 3:**
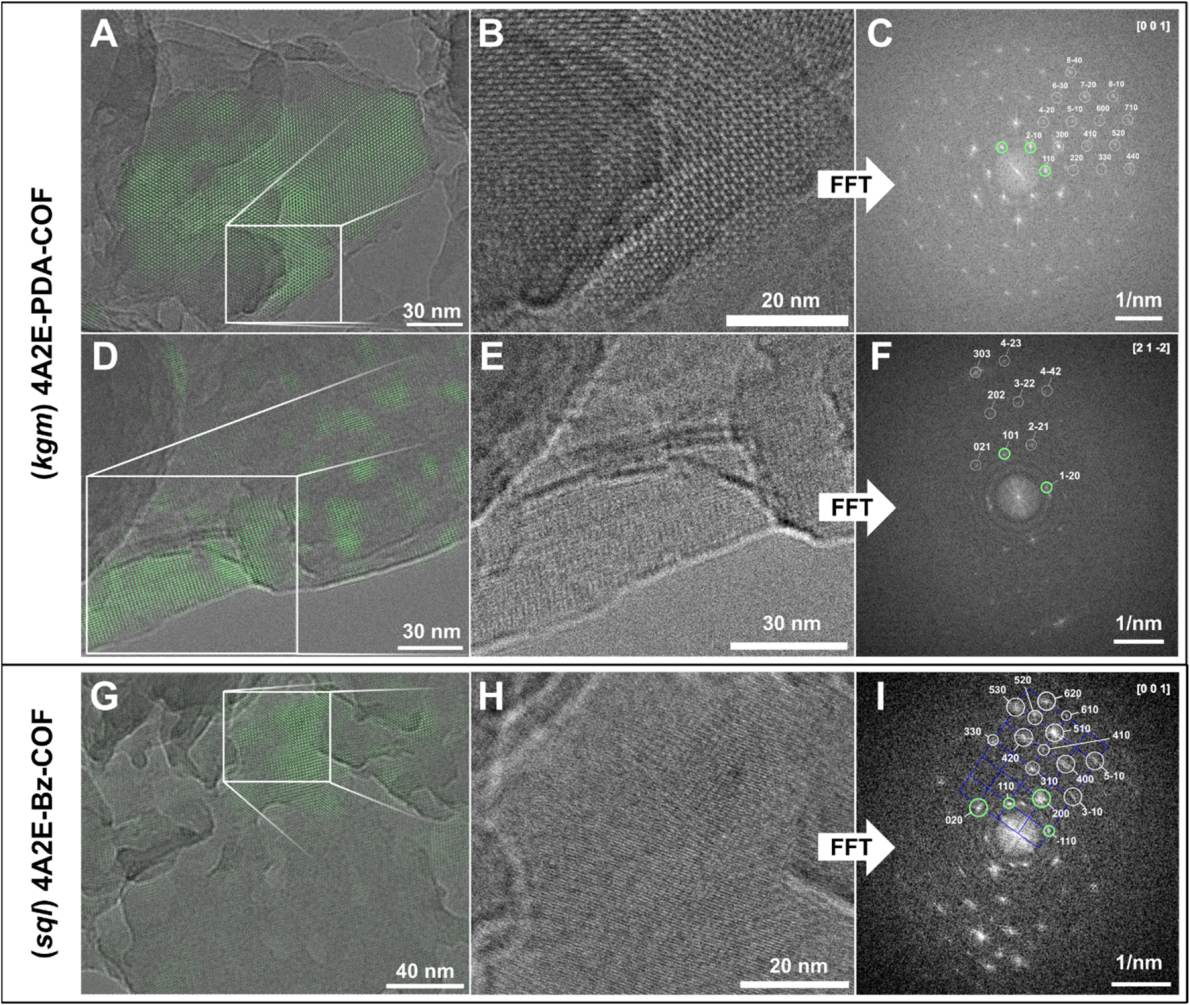
Cryo-TEM images of the 4A2E-PDA-COF (A, B, D and E) and the 4A2E-BZ-COF (G and H) and the corresponding FFT images (C, F and I). The green overlays in A, D and G were generated by the Fourier filtering as indicated in green in the corresponding FFT images (C, F and I).

The PXRD of the benzidine based 4A2E-BZ-COF showed prominent reflexes at 4.60° and 6.44° 2*θ*, which matched well with the *sql* model, therefore corresponding to overlapping reflexes of 110/1–10 and 200/020, respectively (Fig. 2E and F). Pawley refinement led to a good agreement between the observed PXRD and calculated pattern at low and high angles (Fig. 2F). This resulted in a unit cell with lattice parameters of: *a* = 27.5 Å, *b* = 31.9 Å, *c* = 4.4 Å, *α* = 55.0°, *β* = 90°, *γ* = 86.6°. The deviation of *γ* from 90° dramatically improved the Pawley fit and is also supported by TEM images (Fig. 3G–I), which consistently showed crystallites with angles of *γ* = ∼84°. The peak splitting observed in the PXRD at 6.44° indicated a symmetry reduction in the structure,^25^ which was well explained with slip-stacking perpendicular to the 4A2E linker, which leads to the low values of *α* = 55.0°. In *sql* COFs, peak splitting/symmetry reduction can also be explained by *a* ≠ *b* structures,^37^ however refinement of only these parameters with no slip stacking led to chemically unreasonable small values of *a* and *b* during refinement. The Pawley refinements were initially performed using models of a *sql* based structure with a primitive unit cell (*Z* = 1, containing only one formula unit of (4A2E)_1_(BZ)_2_). Lattice parameters and the local symmetry superficially match with the observed crystallites in the TEM (Fig. 3G and H). However, in the FFT additional periodicities can only be explained with a *Z* = 2 superstructure (containing only two formula units: (4A2E)_2_(BZ)_4_), redefined with *a* = (110), *b* = (−110), *c* = (001) respective to the primitive unit cell. The additional periodicities observed in the FFT correspond to the 410, 520 and 610 reflections (Fig. 3I). Supercell periodicities are observed in multiple crystallites in the TEM (Fig. S9 and S10†). We therefore modelled the structure of the 4A2E-BZ-COF as a *Z* = 2 unit cell in Materials Studio.

#### 4A2E-PDA-COF

The PXRD of the 4A2E-PDA-COF showed sharp reflexes with the most intense reflex at 5.22° 2*θ*, with additional less intense reflexes at 7.66°, 9.14°, 12.98°, 14.80°, and 21.40° (Fig. 2B). The simulated *sql* structure pattern clearly does not match the observed PXRD of 4A2E-PDA-COF, as the calculated pattern shows intense reflections at 6.73° and 7.34°, which are not observed experimentally. Surprisingly, the simulated PXRD based on the *kgm* structural model with a *P*3̄ symmetry and eclipsed stacking did not show a good fit either: while some of the reflections match, others are evidently missing. The most intense observed reflection at 5.22° matches well with the 110 reflection of the simulated structure, while the reflection at 10.77° can be explained by the 300 reflection. But crucially, the 100 reflection, predicted by the simulation at 2.98°, is completely absent in the experimentally observed pattern (Fig. 2B) even in small angle measurements (Fig. S12†). Additionally, the observed reflection at 7.66° is absent in the simulated *kgm* pattern. This led us to believe that the *kgm* structural model with eclipsed stacking does not describe the structure accurately.

To gain additional orthogonal information about the crystal structure required for solving the 4A2E-PDA-COF's structure, we performed cryo-TEM. In the TEM the 4A2E-PDA-COF showed intergrown crystallites ranging in size from 100 to 150 nm (Fig. S8†), which rendered single crystal electron diffraction structure determination unfeasible. However, owing to the high resolution achieved in the TEM images, we could observe these crystallites in various orientations, including alignment with the [001] direction, clearly revealing the hexagonal symmetry of the structure (Fig. 3B and C). This observation confirmed the *d*-spacing of 16.9 Å in the fast Fourier transform (FFT) of the TEM images, matching well with the reflection observed in the PXRD at 5.22°, which can be assigned to the 110 reflection in the *kgm* model (Fig. 2B). Additionally, FFT and real space images showed no indications of a 100 reflection anywhere in the TEM images of the 4A2E-PDA-COF (Fig. 3C). This corresponds to the absence of this feature in the PXRD. The observed periodicities in the TEM coupled with the hexagonal symmetry evidently deviate from a simple eclipsed *kgm* model.

The stacking of 2D COFs can lead to symmetry reductions^25^ or even complete changes of a PXRD pattern through the change in space group or symmetry.^33^ Therefore, we considered again the *kgm* structural model, and started building models with different stacking modes, to test if the stacking can explain the absence of the 100 peak in the PXRD and the TEM images. Slip stacking only leads to peak broadening or small peak splitting through apparent symmetry reduction,^25^ but cannot explain the absence of one significant reflection. In AB stacking where one hexagonal pore stacks on top of a triangular pore, while one triangular pore remains open, leads to a dramatic reduction, but not to a complete loss off intensity of the 100 peak in the simulated pattern (Fig. S5†). When simulating the ABC-stacking of the *kgm* net layers of the COF, we obtained a good match with the simulated PXRD pattern (Fig. 2B), which was also validated by Pawley refinement of the ABC-stacked structure (Fig. 2C). Therefore, the lattice parameters are *a* = *b* = 33.49 Å, *c* = 12.31 Å, *α* = *β* = 90°, *γ* = 120.

The ABC stacking leads to a change of the space group to *R*3̄ and systematic absences due to a 3-fold screw axis, which includes the 100 reflection. Additionally, this structural model can explain the previously unexplained reflex at 7.85° 2*θ* (*d* = 11.33 Å), which corresponds to the 101 reflection. The periodicity corresponding to the 101 reflections in the PXRD can also be observed in some crystal orientations in the TEM images (Fig. 3E and F). Explaining the presence of this reflection is important, as in typical hexagonal COFs with small parameters of *c* < 5 Å, no reflections are expected between the “first” and “second” reflection in a hexagonal unit cell, which normally correspond to the 100 and 110 reflections, respectively. In the *R*3̄ space group of the ABC stacked 4A2E-PDA-COF also requires the presence of three layers in one unit cell, each with an interlayer distance of 4.1 Å. While reflections such as 001 are not observed due to systematic absences (only 000*l*: *l* = *3n* are allowed), the 101 reflection is allowed as it fulfils the *hkil*: −*h* + *k* + *l* = 3*n* reflection condition. Therefore, the ABC stacked model of the 4A2E-PDA-COF explains the absent and additional reflections in the PXRD fully, and matches well with the crystallites seen in cryo-TEM.

The ABC-stacking of the *kgm* layers lead to the “closing” of the larger triangular pores with the smaller hexagonal pores with small pore apertures of only around 7 Å, which leads to a pearl-string pore structure, which is quite unusual for COFs (Fig. S4†). The small pore apertures that are present every third layer in the structure, imposed by the six phenyl methoxycarbonyl linkers protruding into the pore and nearly in contact with each other, thereby dominate the adsorption properties and lead to only small micropores being observed in the structure (Fig. S16†). This highly crowded pore is exaggerated by the locking of the imine orientation away from the *ortho*-phenyl ring. This orientation was predicted by force field calculations and verified by the single crystal X-ray diffraction (SCXRD) structure of a model imine compound from aniline and a carboxy dialdehyde linker (Fig. S3†). This orientation can be attributed to steric repulsion with the phenyl ring. Interestingly, the interaction between the imine and the phenyl ring reduces the number of conformers^14^ of the imine bond, which can be used to improve the crystallinity of COFs by forcing it to point away from the phenyl rings. Similar strategies can be realized with intramolecular hydrogen bonding^38^ and ortho methoxy groups.^39^

### Spectroscopic characterization

To further support the above described structural models, we intended to exclude sub-stoichiometric structures, as these can lead to unexpected unit cells and can appear under specific synthesis conditions or when competing interactions are at play in the structure formation.^4,40,41^ When comparing the IR spectra of the 4A2E-linker molecules and the COFs (Fig. S6 and S7†), the strong band corresponding to the C=O stretching of the aldehyde of the 4A2E-linker at 1681 cm^−1^ disappears for the formed COFs, while a vibration at 1612 cm^−1^ and 1616 cm^−1^ appears that can be assigned to the imine C=N stretch vibration for the 4A2E-PDA-COF and 4A2E-Bz-COF, respectively.^42^ The C=O stretch vibration corresponding to the ester group is retained after the formation of the COF, but strongly shifts from 1715 cm^−1^ for the linker to 1733 cm^−1^ and 1729 cm^−1^ for the 4A2E-PDA-COF and 4A2E-Bz-COF, respectively. The characteristic amine vibrations from N–H stretch vibrations in the region 3100–3500 cm^−1^ that are visible in the PDA and benzidine linker completely disappear upon the formation of the COFs.

**Fig. 4:**
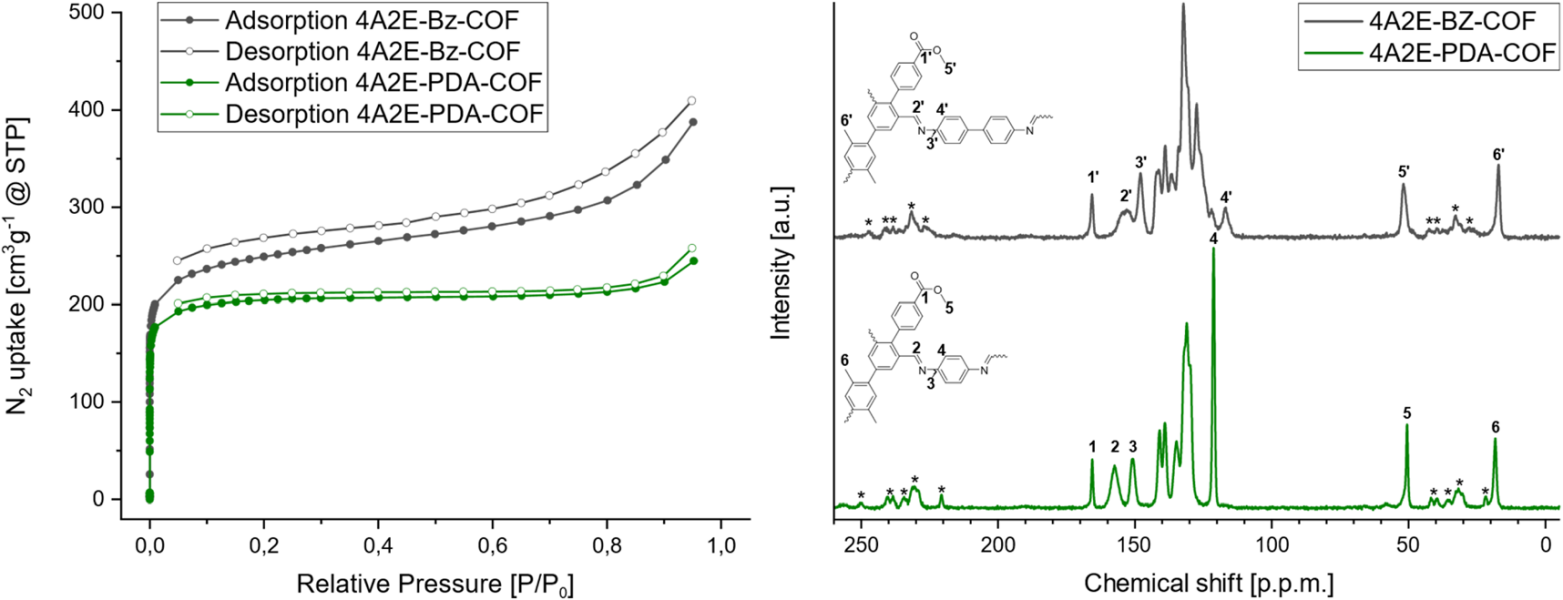
Nitrogen adsorption isotherms at 77 K (left) of 4A2E-PDA-COF (green) and 4A2E-Bz-COF (grey). ^13^C-MAS NMR of 4A2E-PDA-COF (grey) and 4A2E-Bz-COF (green). Asterisks indicate spinning side bands.

The stochiometric nature of the COFs was also verified by solid-state NMR (ssNMR) (Fig. 4B), where no significant aldehyde peak was observed at 190 ppm. At the same time ssNMR showed presence of the imine carbon (2, 2′) which is broadened at 157 ppm and 153 ppm for the 4A2E-PDA-COF and 4A2E-Bz-COF, respectively. The characteristic peak of the quaternary carbon of the ester (1, 1′) at 165 ppm and the methyl carbon of the ester (5, 5′) can be assigned to the peak at 50 ppm and 52 ppm for the 4A2E-PDA-COF and 4A2E-Bz-COF, respectively. The methyl ester peak is thus shifted downfield from the methyl group attached to the 4A2E linker core, which can be found at 18 ppm and 17 ppm for the 4A2E-PDA-COF and 4A2E-Bz-COF, respectively. These measurements strongly indicate a stoichiometric structure as there are no dangling aldehyde groups or amine groups detectable, and an intact methyl ester group. Additionally, dangling amines from a linear diamine would not lead to the formation of a two-periodic structure and would be unlikely, but has been observed before under special circumstances.^43^

#### Sorption

To determine the porosity of the synthesized COFs, nitrogen sorption isotherms were measured at 77 K. The N_2_ gas adsorption isotherms of the 4A2E-PDA-COF showed a type I isotherm with expected steep uptake at low pressures (Fig. 4), as even the “larger” triangular pores in a *kgm* net are below 1.4 nm as seen from the structure simulations. At the same time the “pearl-string” type pores in the ABC stacked structure leads to only a single microporous pore environment. The resulting BET surface showed a moderate area of 804 m^2^ g^−1^ (11 points) using the BETSI software.^44^ This surface area suggests an overall porous structure, despite the narrow pore opening predicted from the structural model. The N_2_-adsorption isotherm of 4A2E-Bz-COF (Fig. 4) showed a slightly higher N_2_ uptake, and an increased BET area of 940 m^2^ g^−1^ (11 points). The pore size distributions were calculated for both materials using the cylindrical pore NLDFT equilibrium model (Fig. S16†). The 4A2E-PDA-COF showed a monomodal pore size distribution with a narrow pore diameter of 1.14 nm, which matches the narrow pore opening of the hexagonal pore. The flexible methyl groups in this pore opening can either point inwards or outwards narrowing or widening the opening. In the open configuration, a diameter of ∼0.96 nm can be measured from carboxyl oxygen to carboxyl oxygen. The 4A2E-Bz-COF showed an increased pore size of 1.6 nm calculated from the adsorption isotherm. The increased size matches with the larger irregularly shaped pore that is generated by the partitioning of the rhombic pore by the methyl benzoate groups, which has a diameter of ∼1.19 nm.

#### Force field simulations

**Fig. 5:**
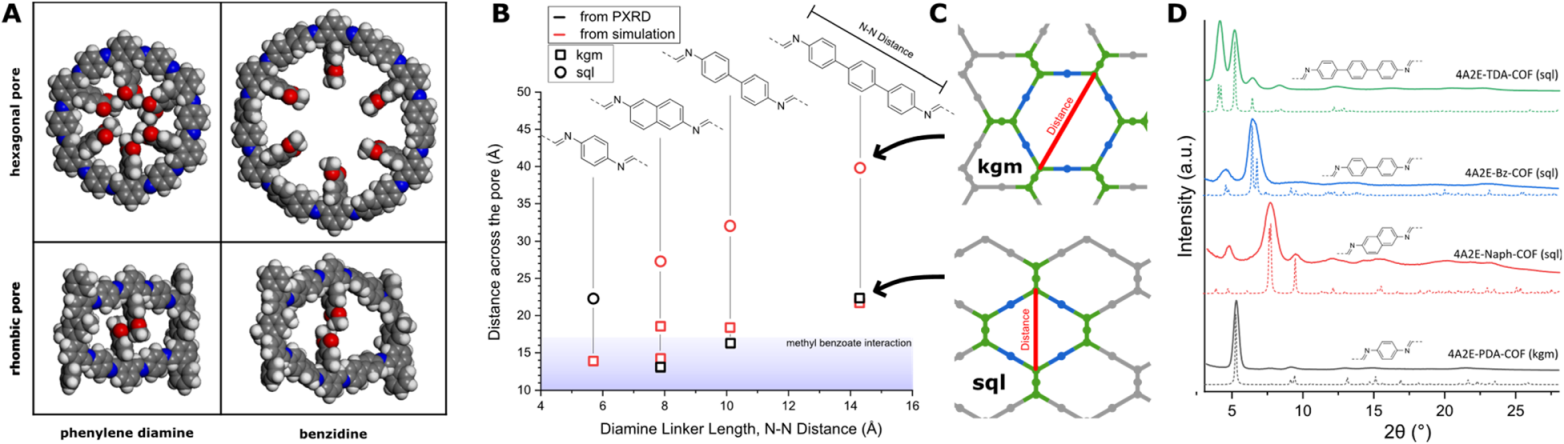
(A) Unconstrained force field simulations of molecular analogues of rhombic and hexagonal pores using PDA and benzidine linkers, all on the same scale. (B) Pore diameters calculated from force field geometry optimized models and PXRD refined structures as a function of the linear linker length. (C) Schematic of the measured pore sizes. (D) Measured (solid line) and calculated (dashed line) PXRD of all four COFs.

To understand the preference for formation of the *kgm* structure over the *sql* structure in the 4A2E-PDA-COF, we used force field simulations to look at the interactions within one pore in unconstrained molecular models (Fig. 5A). In the hexagonal pore the methyl groups are in close contact but no out of plane bending is observed, which would be indicative of an overcrowded pore space. In the case of the rhombic pore and PDA, significant steric repulsion can be observed by the bulky functional groups bending out of each other's way (Fig. 5B). Interestingly, this situation is reinforced by the conformational locking of the imine groups, which forces the bulky functional groups closer to each other. We theorized that this close contact leads to repulsion and thereby disfavours the *sql* structure when using the short phenylenediamine linker. The close contact of the methyl groups across the pore in the *sql* net is directly related to the length of the diamine linker, where a longer diamine might relieve some of the steric repulsion. Simulation of a benzidine based rhombic pore (Fig. 5A) indeed showed less repulsion between the methyl groups despite close contact.

We further investigated this effect by simulating different *sql* and *kgm* unit cells for a set of four linear diamine linkers of varying length (phenylene, naphthyl, biphenyl, terphenyl) together with the 4A2E core without the bulky functional group. The bulky functional groups can interact across the pore in the *sql* and *kgm* structures. Consequently, we measured the distance across the pore for this set of simulated structures each for the *sql* and *kgm* structures (Fig. 5B and C, red symbols). These results show larger pores for the *kgm* structure in comparison to the *sql* structure for each linker. Additionally, the pore size decreases with decreasing length of the linear linker. The possible conformers in this structure are determined by the locked imines as described above and therefore do not play a role in determining the pore size. An exception is the naphthalene based *sql* structure, as the naphthalene unit leads to an offset which can lead to two structural outcomes in the *sql* structure.

To verify these results, we also synthesized the 4A2E-Naph-COF and 4A2E-TAD-COF based on the linkers naphthalene-2,6-diamine (Naph) and 4,4′′-diamino-*p*-terphenyl (TAD), respectively. Both COFs crystallized in the *sql* structure as can be seen from the PXRD (Fig. 5D, solid lines) and their comparison to the simulated patterns based on Pawley refined structures (Fig. 5D, dashed lines, Fig. S13–S16†). Based on these PXRD derived structural models, we measured pore diameters (Fig. 5B, black symbols). Interestingly, these experimentally observed structures show shorter distances across the pore than the simulated pore sizes in the cases where the methyl benzoate functional groups can interact across the pore. This suggests attractive interaction between the functional groups, which narrows the pore. This effect was recently described in other *sql* COFs and termed “wine-rack” motion.^45^ Despite the contraction observed in the naphthalene and benzidine systems, the COF topology switches when transitioning from the naphthalene to the even shorter phenylene linker. This switch indicates that steric interactions become predominantly repulsive when the pore distance becomes too short.

## Discussion

### Controlling the net through steric interactions

Force field calculations of the one pore model indicate that the formation of the hexagonal pore generally leads to a low steric demand of the bulky methyl benzoate functional groups. In the rhombic pore the methyl benzoate groups are much closer to each other (Fig. 5B). This is especially prominent in the case of the short PDA linker. In the case of the longer benzidine, interaction of the methyl benzoate functional groups through the rhombic pore is still evident, but less pronounced than in the PDA case. This indicates that the steric interaction of the bulky functional groups through the pore play a role in determining the formation of the net during the synthesis of the 4A2E-PDA-COF.

Given that all four COFs use the same type of aldehyde node, effects of stacking and reactivity can be excluded,^14^ making steric interaction strength the most significant difference between structures.

The fact that COFs based on naphthalene diamine, benzidine and terphenyl diamine linear linkers all form the *sql* net, strongly suggests that the *sql* net is the “default” or more preferred net for the 4A2E linker. Other COFs reported with similar tetraaldehyde linker cores, but without the bulky functional groups also favour the formation of *sql* COFs for both benzidine and PDA linkers.^46^ The *sql* net is reasonable as “default” net as they generally produce higher density structures and smaller void space per formula unit.^22^

This supports the hypothesis that steric interactions in the PDA-based COF override the preference for the *sql* net through weak interactions, leading to the formation of the *kgm* net instead.

Since the 4A2E linker provides the bulky functional group, while the linear diamine linker determines the strength of the steric interactions, the steric interaction strength can be finely tuned and used to achieve a linker length based topology switch.

### ABC stacked *kgm* COF

The formation of the ABC – stacking of the 4A2E-PDA-COF is a rare example of this stacking type in the *kgm* net. A reason for the ABC stacking can be found in the shape of the 4A2E linker. The linker possesses large dihedral angles along its backbone due to the dimethyl benzene core and the bulky methyl benzoate group next to the aldehyde groups, which inhibit efficient π-stacking of the linker in the resulting COF. This lack of strong interactions between the layers might be responsible for the formation of the ABC stacked structure. This feature of the linker might also be responsible for the slip stacking of the 4A2E-BZ-COF, where the slip stacking relieves unfavourable close contact of the dimethyl benzene core of the linker.

The above described linker features are not particularly unusual, posing the question why the ABC stacking in *kgm* COFs is not more frequently observed. A reason for the apparent rarity of the ABC-stacked *kgm* structures might be the erroneous assignment of a *sql* nets instead of a ABC stacked *kgm* structures. When determining the structure of a COF as either *kgm* or *sql*, the most prominent difference between both models is the reflection with the lowest angle in the PXRD. Here the *kgm* structures produce a reflex at lower angles than a *sql* structure based on the same linkers. However, due to the systematic absence, the first reflex of an ABC *kgm* structure is nearly in the same position as the first reflex of a *sql* structure. In samples with low crystallinity the only possibility to differentiate the structures is through observation of the hexagonal symmetry or square lattice in TEM, which is often not reported. In the COFs reported here, the differentiation between ABC-stacked *kgm* and *sql* is more straightforward, as the methyl benzoate leads to high intensity on reflections at larger angles, and only a very weak reflection at low angles in the *sql* case. This highlights the importance for TEM measurements to not only verify the *d*-spacings, but also the symmetries in the crystallites.

## Conclusions

The toolbox for the COF geometry is mostly limited to the geometry of the linkers. For linker systems where polymorphism is possible, there are no reliable synthesis yet. The linker design strategy described here introduces sterically demanding moieties that can determine the formed nets using through–pore interactions. This shows that additional weak interactions between linkers during the formation of covalent bonds can be a nuanced tool for fine-tuning the obtained structures. In the reported COFs, the steric interactions strength can be directly modulated by varying the length of the linear diamine, yielding a straightforward method for controlling the net. Instead of the repulsive through-pore interactions observed here, also attractive interactions can be imagined that would lead to a reversed trend.

This work also shows that steric interactions need to be considered when designing highly functional COFs, as the side chains in COFs should not be neglected and can influence the structure significantly. Therefore, the weak interactions might play a role in structure formation of a wide range of COFs.

## Author contributions

Alena Winter: investigation, methodology, writing – original draft, writing – reviewing and editing; Farzad Hamdi: investigation, writing – reviewing and editing; Andreas Eichhöfer: investigation; Kay Saalwächter: investigation, writing – reviewing and editing; Panagiotis L. Kastritis: investigation, writing – reviewing and editing; Frederik Haase: conceptualization, methodology, writing – original draft, writing – reviewing and editing, supervision, funding acquisition.

## Conflicts of interest

There are no conflicts to declare.

## Supplementary Material

SC-015-D4SC03461A-s001

SC-015-D4SC03461A-s002

## Data Availability

Additional experimental details, NMR spectra, crystallographic details can be found in the ESI.† The authors have cited additional references within the ESI.†^47–51^ The crystal structures of intermediate 5 and the model compound methyl 2′,6′-bis[(phenylimino)methyl][1,1′-biphenyl]-4-carboxylate have been deposited in the Cambridge Crystallographic Data Centre under the deposition numbers CCDC-2312146 and CCDC-2312147, respectively.
