# Supplementary material for: Enhancing structural control in covalent organic frameworks through steric interaction-driven linker design
Source: Chem Sci. 2024 Aug 13;15(35):14449–57. doi: 10.1039/d4sc03461a (PMC11331305; doi:10.1039/d4sc03461a)
Supplement: SC-015-D4SC03461A-s001 [file SC-015-D4SC03461A-s001.pdf]

Supplementary Information (SI) for Chemical Science.
This journal is © The Royal Society of Chemistry 2024

Supplementary information
for

Enhancing Structural Control in Covalent Organic Frameworks through Steric Interaction-Driven Linker Design

Alena Winter, Farzad Hamdi, Andreas Eichhöfer, Kay Saalwächter, Panagiotis Kastritis, Frederik Haase*

Table of Contents

Linker Synthesis 2
Model compound synthesis 4
COF synthesis 5
Methods 6
Supplemental Figures 8
Supplementary References 16

Linker Synthesis

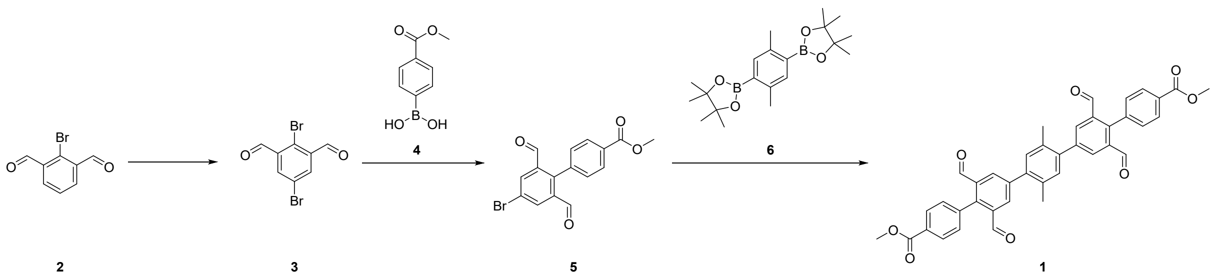

Figure S 1: Synthesis strategy of the **4A2E** linker.

2,5-Dibromoisophthalaldehyde (3)[1]

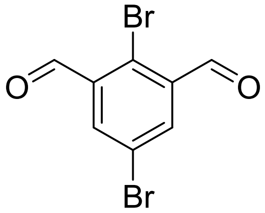

In a 50 mL crimp vial, 2-bromoisophthalaldehyde (5.00 g, 23.5 mmol, 1.00 equiv.) and *N*-bromosuccinimide (5.00 g, 28.1 mmol, 1.20 equiv.) was dissolved in concentrated sulfuric acid (25 mL) and stirred at 85 °C for 16 h. The solution was poured into a solution of a stoichiometric amount of sodium bicarbonate in water and the product was extracted several times into dichloromethane (DCM). The solvent was removed under reduced pressure and the residue was purified by flash chromatography (CH/DCM; 1:1). The product was obtained as an off-white solid in a yield of 69% (4.76 g).

^{1}H NMR (400 MHz, $CDCl_3$) δ [ppm] = 10.46 (s, 2 H), 8.22 (s, 2 H)

^{13}C NMR (100 MHz, $CDCl_3$) δ [ppm] = 189.31, 137.80, 135.83, 128.76, 123.24

Methyl 4'-bromo-2',6'-diformyl-[1,1'-biphenyl]-4-carboxylate (4)

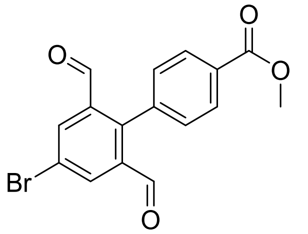

In a 100 mL 3-neck flask, 2,5-dibromoisophthalaldehyde (1.00 g, 3.42 mmol, 1.25 equiv.) and (4-methoxycarbonylphenyl)boronic acid (493 mg, 2.74 mmol, 1.00 equiv.) were dissolved in dry 1,4-dioxane (15 mL) and bubbled for 20 min. Pd(dppf)Cl_2 (100 mg, 137 µmol, 5 mol%) was added and the solution was bubbled for additional 10 min. K_3PO_4 (1.74 g, 8.22 mmol, 3.00 equiv.) was dissolved in water (5 mL) and degassed for 10 min. Afterwards it was added to the reaction mixture. The reaction was stirred at 90 °C for 16 h. After cooling to room temperature, the reaction mixture was filtered and the solvents were removed under reduced pressure. The precipitate was dissolved in DCM (40 mL) and washed with water (3 * 50 mL) and brine (1 * 50 mL). The organic phase was dried over Na_2SO_4 and evaporated under reduced pressure. The crude residue was purified via flash column chromatography (CH:EtOAc). A higher catalyst concentration leads to dehalogenation reaction. Yield: 599 mg (63%, 1.73 mmol)

^{1}H NMR (400 MHz, $CDCl_3$) δ [ppm] = 9.71 (s, 2 H), 8.36 (s, 2 H), 8.21 (d, J = 8.5 Hz, 2 H), 7.47 (d, J = 8.5 Hz, 2 H), 3.99 (s, 3 H)

^{13}C NMR (100 MHz, $CDCl_3$) δ [ppm] = 188.9, 166.3, 145.2, 136.5, 136.2, 135.6, 131.4, 130.9, 130.0, 124.0, 52.7

The substitution pattern was also verified via X-ray structure analysis of a single crystal grown by slow evaporation of $CDCl_3$. The crystal structure has been deposited to the CCDC under the number: CCDC-2312146.

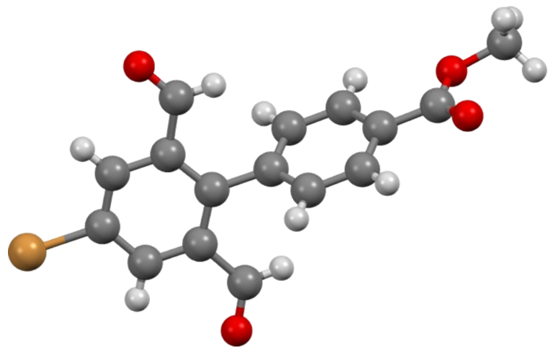

Figure S 2: 3D structure of Methyl 4'-bromo-2',6'-diformyl-[1,1'-biphenyl]-4-carboxylate confirming the cross coupling at the 1’ position.

1,4-Bis(pinacolatoboronyl)-2,5-dimethylbenzene (6)

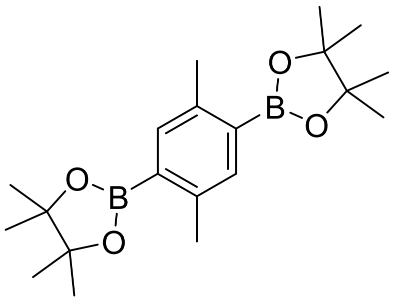

Following a literature procedure[2], in a 50 mL crimp vial under a nitrogen atmosphere, 2,5-dibromo-*p*-xylene (1.06 g, 4.00 mmol, 1.00 equiv.), B_2pin_2 (3.05 g, 12.0 mmol, 3.00 equiv.), Pd(dppf)Cl_2 (0.196 g, 0.24 mmol, 0.06 equiv.), and KOAc (2.36 g, 24.0 mmol, 6.00 equiv.) were dissolved in dry DMF(40 mL) and degassed with N_2 for 20 min. The reaction mixture was heated at 85 °C for 48 h. The reaction mixture was cooled down to room temperature, then added to water before being extracted with DCM (50 mL). Combined organic layers were washed with water (2 * 100 mL) and brine (100 mL), then dried with Na2SO4 and evaporated under reduced pressure. The crude product was purified by recrystallization in MeOH. The product was obtained as a beige solid in a yield of 77% (1.11 g)

^{1}H NMR (400 MHz, $CDCl_3$) δ [ppm] = 7.53 (s, 2 H), 2.48 (s, 6 H), 1.34 (s, 24 H)

^{13}C NMR (100 MHz, $CDCl_3$) δ [ppm] = 140.69, 137.05, 83.55, 25.03, 21.62

Dimethyl 2',3''',5''',6'-tetraformyl-2'',5''-dimethyl-[1,1':4',1'':4'',1''':4''',1''''-quinquephenyl]-4,4''''-dicarboxylate (4A2E)

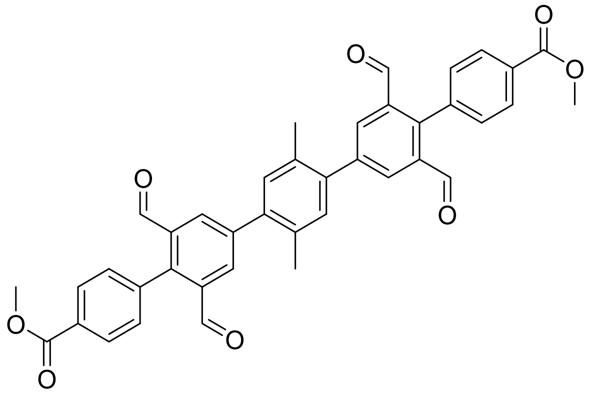

In a 100 mL 3-neck flask under a nitrogen atmosphere, methyl 4'-bromo-2',6'-diformyl-[1,1'-biphenyl]-4-carboxylate (882 mg, 2.54 mmol, 2.50 equiv:), 1,4-Bis(pinacolatoboronyl)-2,5-dimethylbenzene (362 mg, 1.01 mmol, 1.00 equiv:) and $Pd(PPh_3)_4$ (133 mg, 115 µmol, 0.10 equiv) were dissolved in a mixture of dry toluene and dry 1,4-dioxane (1:1; 16 mL) and degassed with N_2 for 15 min. K_2CO_3 (796 mg, 5.76 mmol, 5.00 equiv.), dissolved in water (4 mL), was degassed for 10 min and added to the reaction mixture. The reaction was heated to 110 °C for 16 h. After the reaction cooled down to room temperature, the insoluble precipitate was filtered off and washed with EtOAc and water and dissolved in DCM (White solid). To increase the yield, the organic phase of the reaction mixture was washed with water (2 * 20 mL). After phase separation, the aqueous phase was extracted with DCM (3* 20 mL). The combined organic extracts were dried over Na_2SO_4 and the solvent was removed under reduced pressure. The crude material was purified *via* column chromatography of silica gel with CH/DCM as an eluent.

Yield: 477 mg (74%, 0.747 mmol)

^{1}H NMR (400 MHz, $CDCl_3$) δ [ppm] = 9.86 (s, 4 H), 8.30 (s, 4 H), 8.25 (d, J = 8.5 Hz, 4 H), 7.56 (d, J = 8.5 Hz, 4 H), 7.27 (s, 2 H), 4.01 (s, 6 H), 2.36 (s, 6 H)

^{13}C NMR (100 MHz, $CDCl_3$) δ [ppm] = 190.19, 166.25, 145.23, 142.36, 139.07, 137.29, 134.61, 133.28, 133.15, 132.14,130.99, 130.95, 129.74, 42.48, 19.84

Model compound synthesis

Methyl 2',6'-diformyl-[1,1'-biphenyl]-4-carboxylate

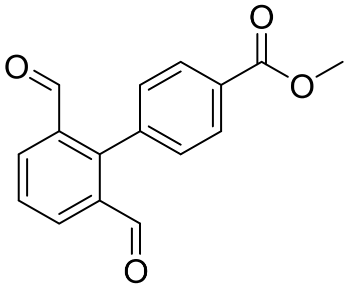

Under nitrogen atmosphere in a 50 mL crimp vial, 2-bromobenzene-1,3-dicarbaldehyde (2.43 g, 11.4 mmol, 1.00 equiv), (4-methoxycarbonylphenyl)boronic acid (3.08 g, 17.1 mmol, 1.50 equiv), potassium carbonate (4.73 g, 34.2 mmol, 3.00 equiv) and dichloropalladium;triphenylphosphane (401 mg, 571 µmol, 0.0500 equiv) were dissolved in dry 1,4-dioxane (20.0 mL) and degassed for

10 min. Degassed water (5.00 mL) was added and the reaction was heated to 100 °C for 4 h. The reaction mixture was cooled down, diluted with DCM and washed with water and brine. Difficulties in the phase separation. The organic phase was dried over $MgSO_4$. The crude residue was was purified via flash column chromatography (30% DCM in CH to 100% DCM). Yield: 1.35 g (44%, 5.02 mmol)

^{1}H NMR (400 MHz, $CDCl_3$) δ [ppm] = 9.78 (s, 2 H), 8.27 (d, J = 7.7 Hz, 2 H), 8.21 (d, J = 8.2 Hz, 2 H), 7.71 (t, J = 7.7 Hz , 1H) 7.48 (d, J = 8.2 Hz, 2 H), 3.99 (s, 3 H)

^{13}C NMR (100 MHz, $CDCl_3$) δ [ppm] = 188.8, 166.2, 145.1, 136.4, 136.1, 135.5, 131.3, 130.9, 129.9, 123.9, 52.6

Methyl 2',6'-bis[(phenylimino)methyl][1,1'-biphenyl]-4-carboxylate

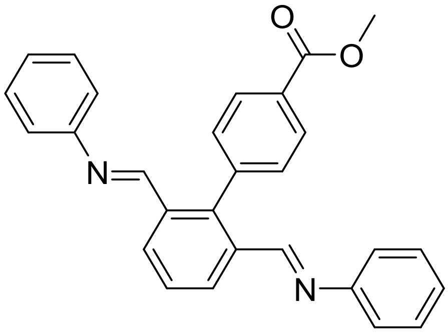

Methyl 2',6'-diformyl-[1,1'-biphenyl]-4-carboxylate (25.0 mg, 93.2 µmol, 1.00 equiv) and aniline (17.0 µL, 17.4 mg, 186 µmol, 2.00 equiv) were dissolved in deuterated chloroform (500 µL). The reaction mixture was stirred at 60 °C for 4 h. Crystals were grown by slow evaporation of CDCl3. The structure was verified by single crystal X-ray analysis and has been deposited to the CCDC under the number: CCDC- 2312147.

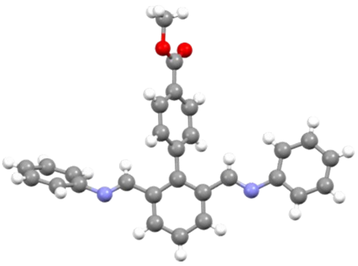

Figure S 3: 3D structure of Methyl 2',6'-bis[(phenylimino)methyl][1,1'-biphenyl]-4-carboxylate showing the imine-nitrogen pointing away from the ortho phenyl ring.

COF synthesis

4A2E-PDA-COF: A 50 mL vial was charged with tetraaldehyde linker (59.4 mg, 0.093 mmol, 1.00 equiv.). It was suspended in a mixture 1,4-dioxane/mesitylene (550 µL:4.4 mL). TFA (114.6 µL, 170.6 mg, 1.50 mmol, 16.0 equiv.) was added and subsequently phenylenediamine (20.19 mg, 0.187 mmol, 2.00 equiv., dissolved in 550 µL 1,4-dioxane) was added. The vial was closed and placed in the ultrasonic bath for 10 min. The mixture was heated in an oven at 120 °C for 3 days. After being cooled to room temperature, the solvent was filtered of and the solid was washed thoroughly with methanol. Soxhlet extraction with MeOH overnight followed by supercritical CO_2 drying yielded PDA-COF as a yellow solid.

4A2E-Bz-COF: A 50 mL vial was charged with tetraaldehyde linker (59.75 mg, 0.0935 mmol, 1.00 equiv.). It was suspended in a mixture 1,4-dioxane/mesitylene (1.1 mL:4.4 mL). TFA (453.2 µL, 674.81 mg, 5.92 mmol, 63.3 equiv.) was added and subsequently benzidine (34.45 mg, 0.187 mmol, 2.00 equiv.) was added. The vial was closed and placed in the ultrasonic bath for 10 min. The mixture was heated in an oven at 120 °C for 3 days. After being cooled to room temperature, the solvent was filtered of and the solid was washed thoroughly with methanol. Soxhlet extraction with MeOH overnight followed by supercritical CO_2 drying yielded BZ-COF as a yellow solid.

4A2E-Naph-COF: A 0.5 mL – 2.0 mL Biotage® microwave reaction vial was charged with tetraaldehyde linker (10.86 mg, 0.017 mmol, 1.00 equiv.). It was suspended in a mixture of mixture 1,4-dioxane/mesitylene (200 µL:800 µL). TFA (82.4 µL, 122.7 mg, 1.08 mmol, 63.5 equiv.) was added and subsequently naphthalene-2,6-diamine (5.38 mg, 0.034 mmol, 2.00 equiv.) was added. The vial was closed and placed in the ultrasonic bath for 10 min. The mixture was heated in an oven at 120 °C for 3 days. After being cooled to room temperature, the solvent was filtered of and the solid was washed thoroughly with methanol. Soxhlet extraction with MeOH overnight followed by supercritical CO_2 drying yielded Naph-COF as a green solid.

4A2E-TAD-COF: A 0.5 mL – 2.0 mL Biotage® microwave reaction vial was charged with tetraaldehyde linker (10.86 mg, 0.017 mmol, 1.00 equiv.). It was suspended in a mixture of mixture 1,4-dioxane/mesitylene (500 µL:500 µL). TFA (82.4 µL, 122.7 mg, 1.08 mmol, 63.5 equiv.) was added and subsequently 4,4''-diamino-*p*-terphenyl (8.85 mg, 0.034 mmol, 2.00 equiv.) was added. The vial was closed and placed in the ultrasonic bath for 10 min. The mixture was heated in an oven at 120 °C for 3 days. After being cooled to room temperature, the solvent was filtered of and the solid was washed thoroughly with methanol. Soxhlet extraction with MeOH overnight followed by supercritical CO_2 drying yielded TAD-COF as a khaki solid.

Methods

Materials and methods

All materials were obtained from commercial sources (TCI, BLD Pharm, abcr, Sigma Aldrich, FisherScientific/ThermoFisher)

NMR: The solution state NMR spectra were acquired using an Agilent Technologies 400 MHz VNMRS and 500 MHz DD2 spectrometer at a temperature of 27 °C. The chemical shifts (denoted as δ) are presented in parts per million (ppm) and are calibrated with respect to the residual signal of the solvent ($CDCl_3$: 7.26 ppm for ^1H and 77.16 ppm for ^{13}C)

ssNMR: The ^{13}C CP-MAS spectra were obtained on a Bruker Avance III spectrometer with proton resonance frequency 400 MHz. The samples were packed in 4 mm rotors and spun at MAS rate 10 kHz at ambient temperature. Experimental parameters: CP contact time 2.5 ms, ^1H 90-degree pulse 3.6 microseconds; the ^{13}C signal was recorded during 35 ms with 65 kHz SPINAL proton decoupling.

IR: Measurements were conducted using a DiaMaxATR unit (Harrik, Pleasantville, NY, USA), which was integrated with a Tensor 27 spectrometer (BRUKER, Billerica, USA). The sample was applied onto the ATR crystal and compressed using the pressure applicator, featuring a force-limited slip-clutch mechanism. The experiment was carried out at room temperature and consisted of 256 accumulations, with data acquisition managed by the OPUS software. Subsequently, background-corrected spectra were analyzed using the OPUS software and further processed in Origin.

N_2-sorption: All N_2-sorption measurements were carried out using a Quantachrome autosorb iQ2. Before gas adsorption measurements, the sample was activated by drying under a vacuum at 120 °C for 16 h. The resulting sample was then used for gas adsorption measurements from 0 to 1 atm at 77 K.

The Brunauer-Emmett-Teller (BET) method was utilized to calculate the specific surface areas using the BETSI software[3] using 11 datapoints for each material. Pore sizes were calculated using the N_2 at 77 K on silica (cylindr. pore. NLDFT equilibrium model).

PXRD: Measurements were conducted with an Incoatec (Geesthacht, Germany) IµS equipped with a microfocus source and a monochromator for CuKα radiation (l=1.54 Å). The 2D scattering patterns were captured using a Vantec 500 2D detector (Bruker AXS, Karlsruhe). Moving the detector allowed acquisition of diffractograms spanning both small and wide angles.

TEM: The COF sample was suspended in 1-butanol at a concentration of 2 mg/mL. This suspension underwent an 8-minute sonication process at room temperature in a standard ultrasonic bath (Bandelin Sonorex Digiplus - Bandelin Electronic GmbH & Co. KG) and was subsequently centrifuged at 17,000 g for 3 minutes (Heraeus Fresco 21 - ThermoFisher Scientific). Following this, a 3.5 µl aliquot of the supernatant 1-butanol was dispensed onto the carbon side of lacey grids, which were already laid on 525-type ashless filter paper to remove excess solution. This technique enables the lacey film to selectively filter fine COF particles. Subsequently, the grids were air-dried and then securely affixed within the ThermoFisher Autogrid assembly using the standard tools, all conducted at room temperature.

The prepared samples were loaded into a Thermo Fisher Scientific Glacios cryogenic electron microscope, operating at 200 kV and equipped with a Falcon 4i direct electron detector. These samples were left to equilibrate overnight under vacuum conditions within the microscope's autoloader at room temperature to ensure thorough drying. Following this, the samples were gradually cooled to cryogenic temperatures (below 100 °K) inside the microscope while maintaining the vacuum.

For imaging, a low-dose strategy was employed. Images were captured with an average electron dose of 50 e/Å² at a pixel size of 0.936 Å in electron counting mode, and a frame rate of 310/s. The electron event data underwent motion correction and were stored as a single frame using the on-the-fly frame alignment feature of the Falcon 4i camera.

Single Crystal X-ray Analysis: Diffraction data of Methyl 4'-bromo-2',6'-diformyl-[1,1'-biphenyl]-4-carboxylate (**4**) and Methyl 2′,6′-bis[(phenylimino)methyl][1,1′-biphenyl]-4-carboxylate were collected on a STOE STADI VARI diffractometer with monochromated Mo Kα (0.71073 Å) or Ga Kα (1.34143 Å) radiation at low temperature. Using Olex2[4], the structures were solved with the ShelXT[5] structure solution program using Intrinsic Phasing and refined with the ShelXL[6] refinement package using Least Squares minimization. Refinement was performed with anisotropic temperature factors for all non-hydrogen atoms; hydrogen atoms were calculated on idealized positions. Crystallographic data and refinement details are summarized in Table S1.

Table S1 Crystallographic data and refinement details.

Compound	Methyl 4'-bromo-2',6'-diformyl-[1,1'-biphenyl]-4-carboxylate (**4**)	Methyl 2′,6′-bis[(phenylimino)methyl][1,1′-biphenyl]-4-carboxylate
Empirical formula	$C_{16}H_{11}BrO_4$	$C_{28}H_{22}N_2O_2$
Formula weight	347.16	418.47
Temperature/K	180.0	180
Crystal system	orthorhombic	triclinic
Space group	$Pna2_1$	$P1$
a/Å	14.3243(10)	9.9945(16)
b/Å	11.4688(7)	10.1084(12)
c/Å	8.7762(8)	11.4916(15)
α/°	90	82.539(10)

β/°	90	73.454(11)
γ/°	90	76.360(11)
Volume/Å^3	1441.78(19)	1079.1(3)
Z	4	2
ρ_{calc}g/cm^3	1.599	1.288
μ/mm^{-1}	2.757	0.082
F(000)	696.0	440.0
Crystal size/mm^3	0.08 × 0.058 × 0.015	0.22 × 0.2 × 0.18
Radiation	Ga Kα (λ = 1.34143)	Mo Kα (λ = 0.71073)
2Θ range for data collection/°	12.29–128.0	3.71–52.0
Index ranges	$-18 \le h \le 17$,<br>$-15 \le k \le 11$,<br>$-11 \le l \le 9$	$-12 \le h \le 12$,<br>$-12 \le k \le 9$,<br>$-14 \le l \le 14$
Reflections collected	9338	8450
Independent reflections	2842 [R_{int} = 0.0630]	4194 [R_{int} = 0.1187]
Ind. refl. with I≥2σ (I)	2277	2078
Data/restraints/parameters	2842/1/191	4194/0/290
Goodness-of-fit on F^2	1.031	0.973
Final R indexes [I≥2σ (I)]	R_1 = 0.0383, wR_2 = 0.0973	R_1 = 0.1171, wR_2 = 0.2769
Final R indexes [all data]	R_1 = 0.0485, wR_2 = 0.1020	R_1 = 0.1826, wR_2 = 0.3287
Largest diff. peak/hole / e Å^{-3}	0.40/–0.52	0.55/–0.73
Flack parameter	–0.05(3)	
CCDC number	2312146	2312147

Supplemental Figures

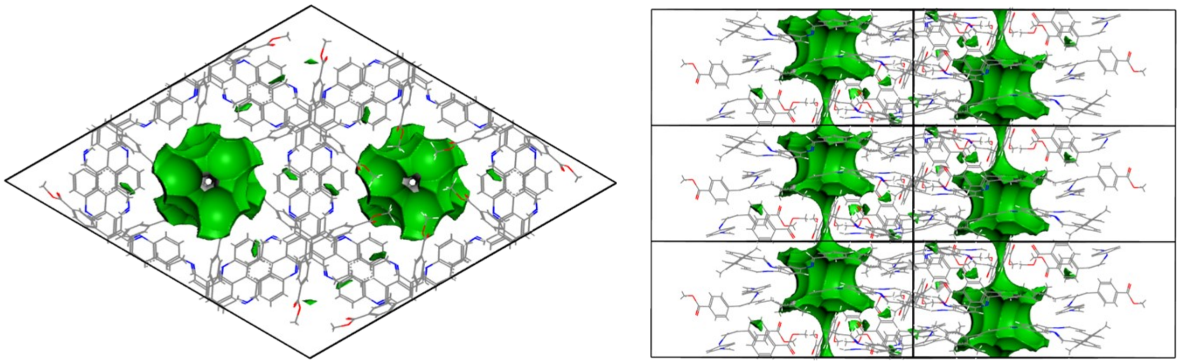

Figure S 4: Solvent accessible surfaces of the two central pores in the **4A2E**-PDA-COF with a 1.4 Å Probe radius viewed along the [001] direction (left) and the [110] direction (right). Symmetry equivalent pores at the unit cell boundary were omitted for clarity.

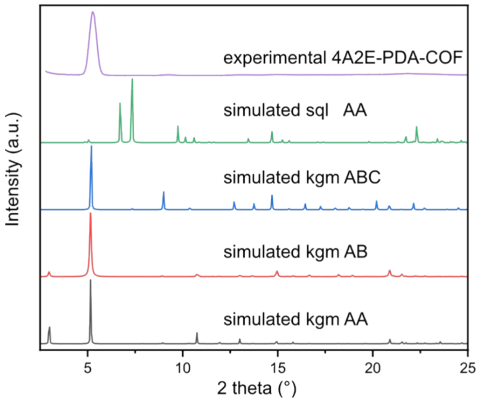

Figure S 5: PXRD comparison **4A2E**-PDA-COF with *sql* AA, *kgm* ABC, *kgm* AB and *kgm* AA stacking.

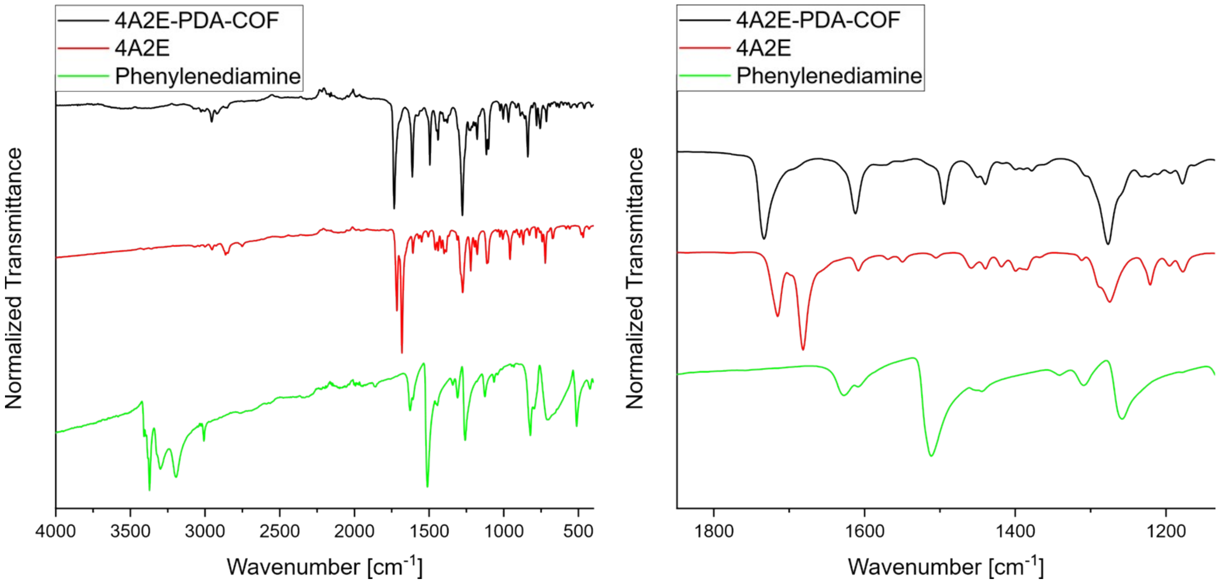

Figure S 6: IR of **4A2E**-PDA-COF (black), **4A2E** linker (red) and Phenylenediamine (green).

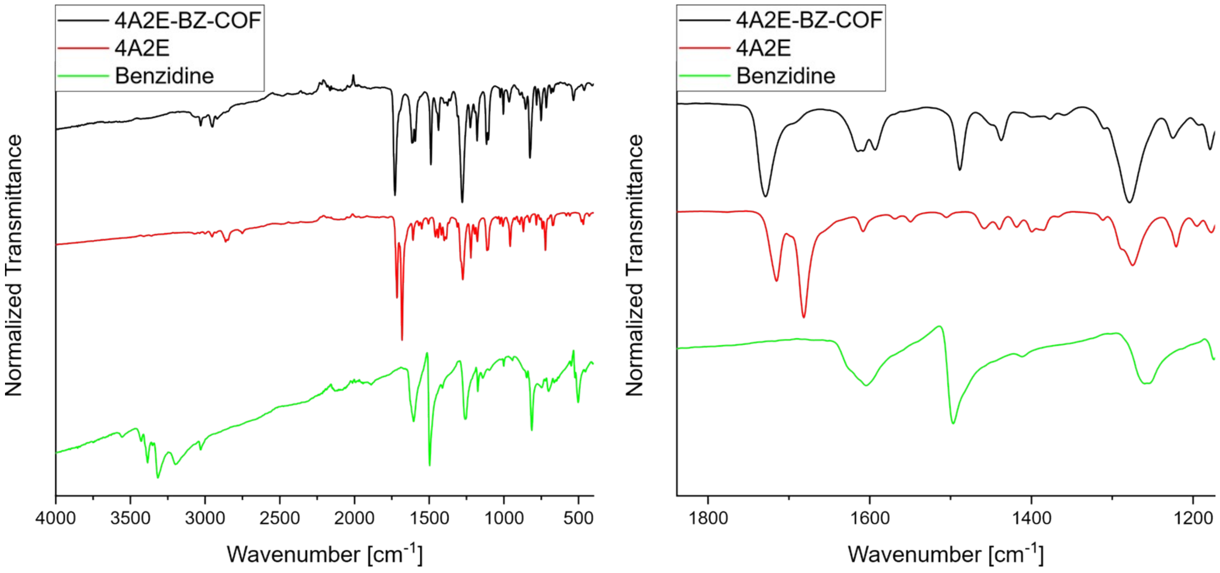

Figure S 7: IR spectra of **4A2E**-BZ-COF (black), **4A2E** linker (red) and Benzidine (green).

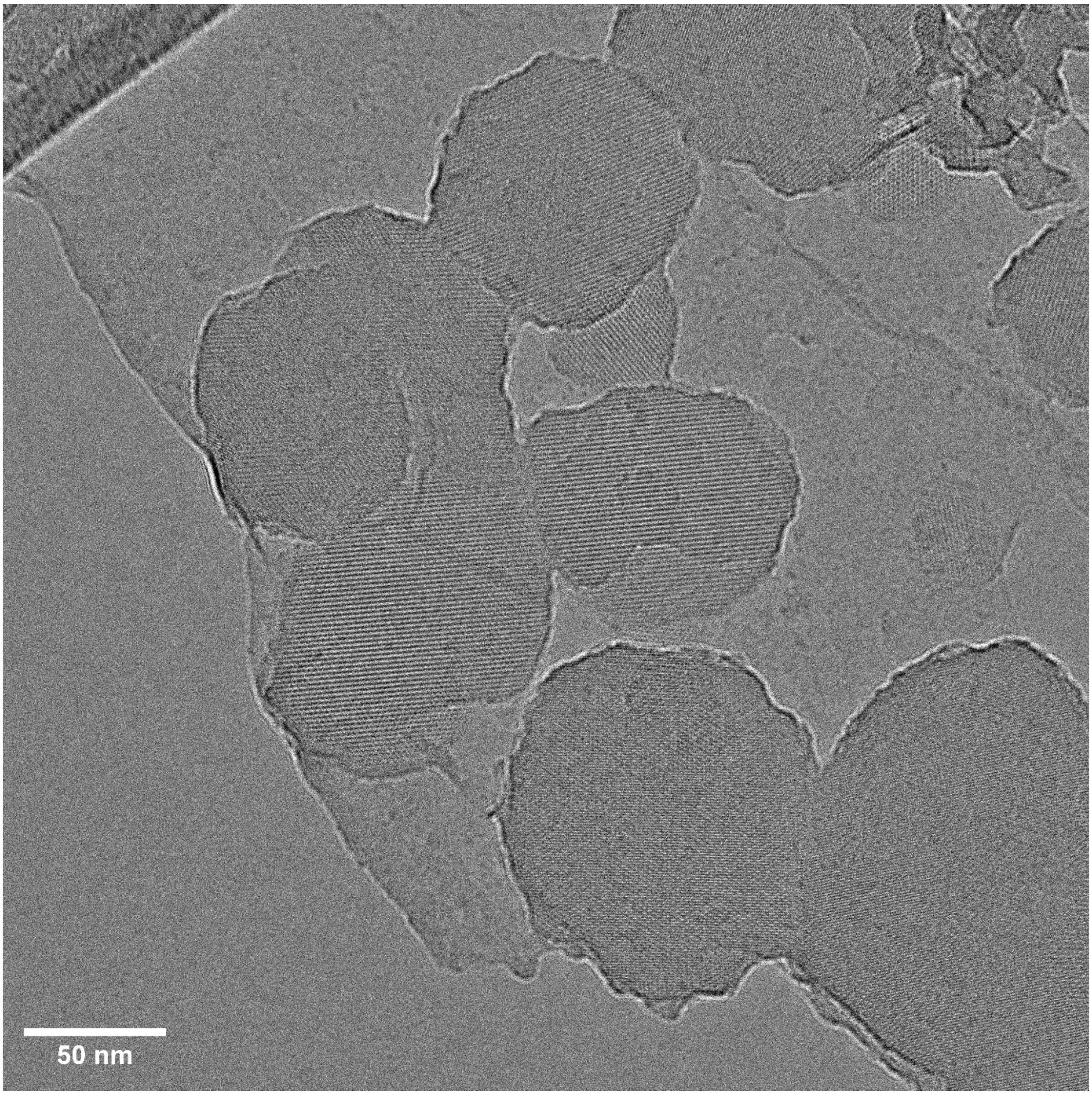

Figure S 8: Overview of particles of the **4A2E**-PDA-COF

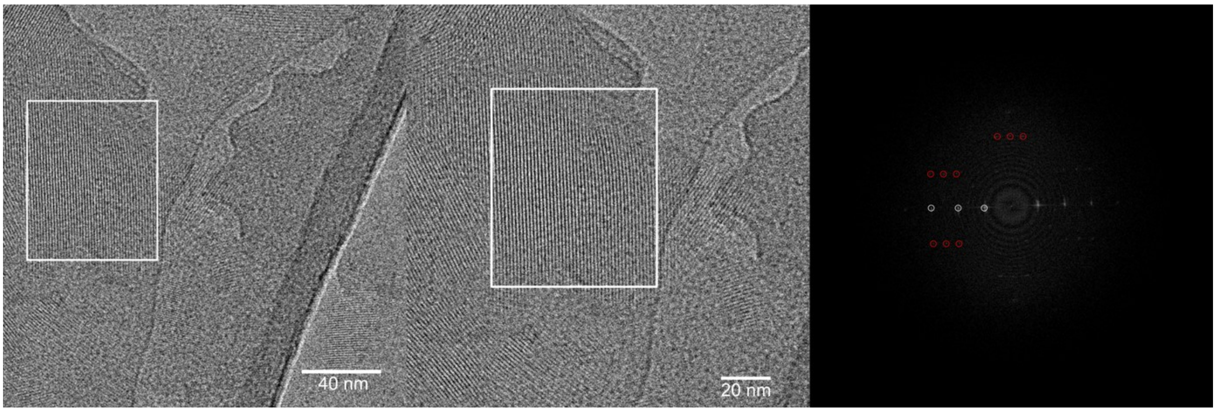

Figure S 9: TEM images of the **4A2E**-Bz-COF (left, middle) and the corresponding FFT image (right). The red circles in the FFT represent the superstructure.

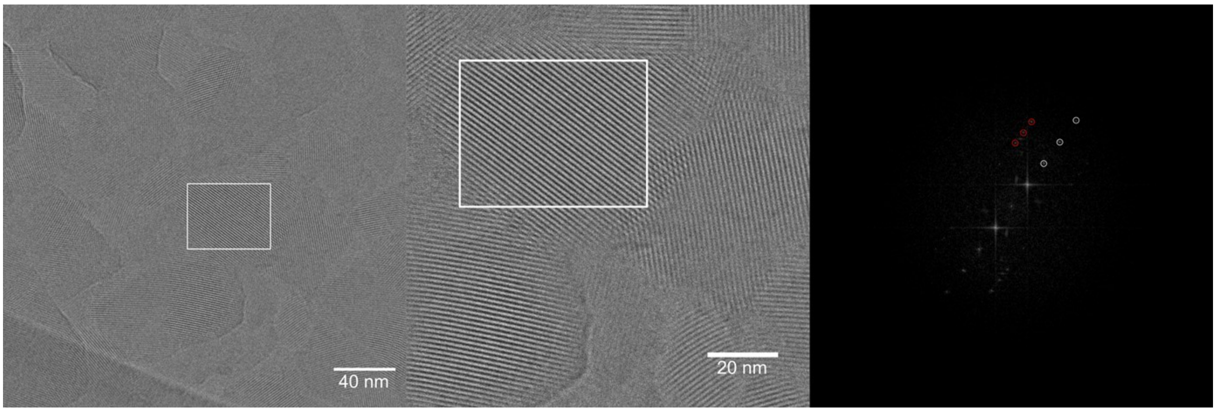

Figure S 10: TEM images of the **4A2E**-Bz-COF (left, middle) and the corresponding FFT image (right). The red circles in the FFT represent the superstructure.

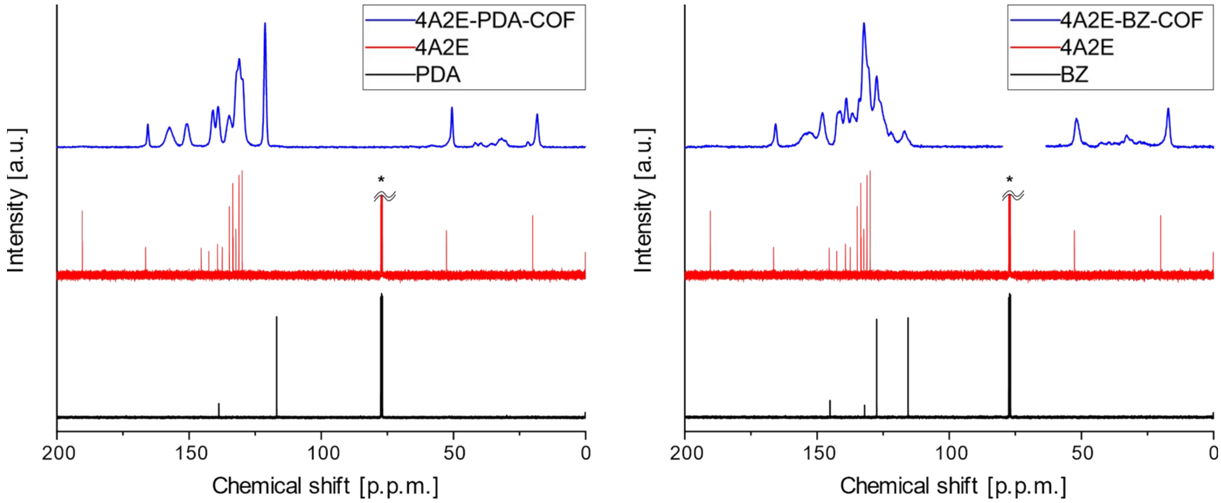

Figure S 11: Comparison of ssNMR of COFs (blue) with solution state NMR of **4A2E** (red) and diamine (black). **4A2E**-PDA-COF (left) **4A2E**-BZ_COF (right).

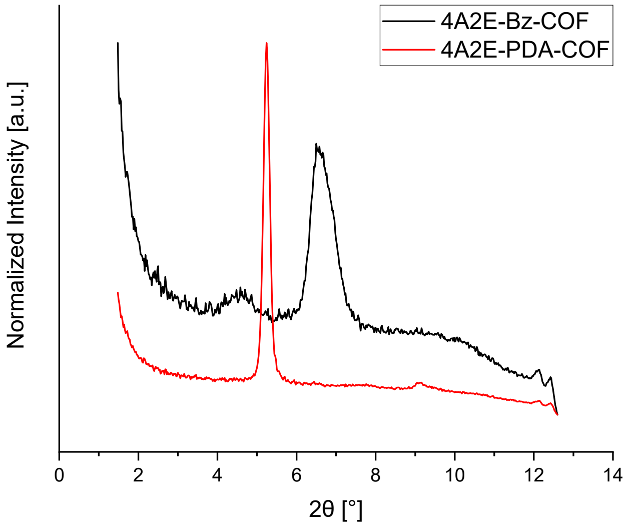

Figure S 12: PXRD measurement of the **4A2E**-PDA-COF and **4A2E**-Bz-COF in the small angle configuration.

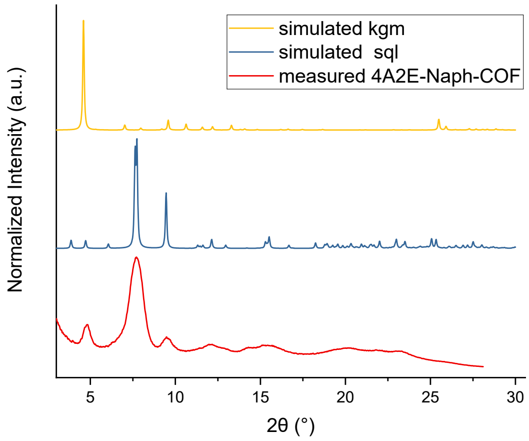

Figure S 13: PXRD measurement of the 4A2E-Naph-COF in wide angle configuration. Comparison with simulated *kgm* and *sql* PXRD spectra.

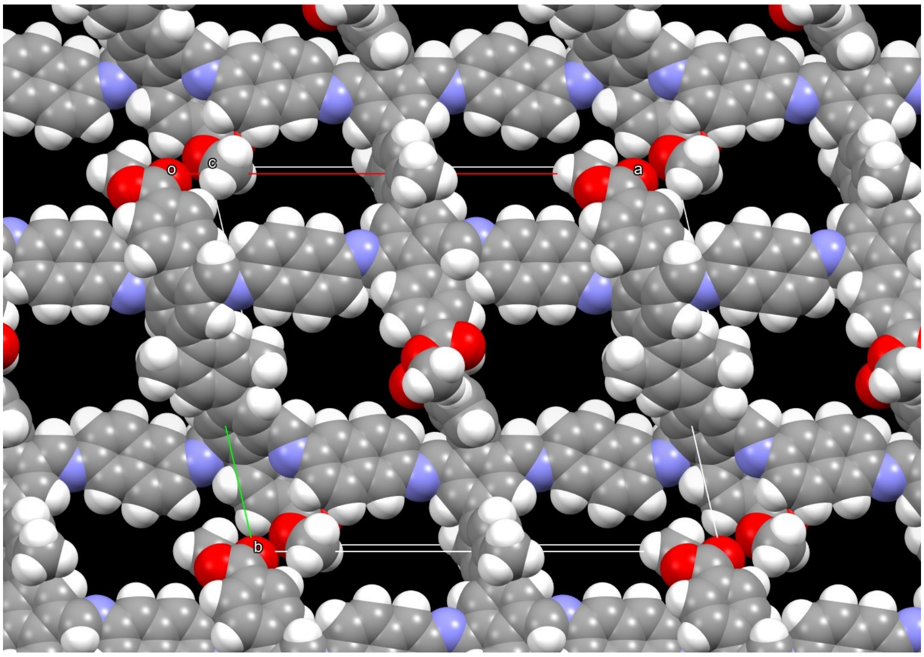

Figure S 14: Depiction of the 2,6-Naphthalenediamine based 4A2E-Naph-COF in the *sql* network.

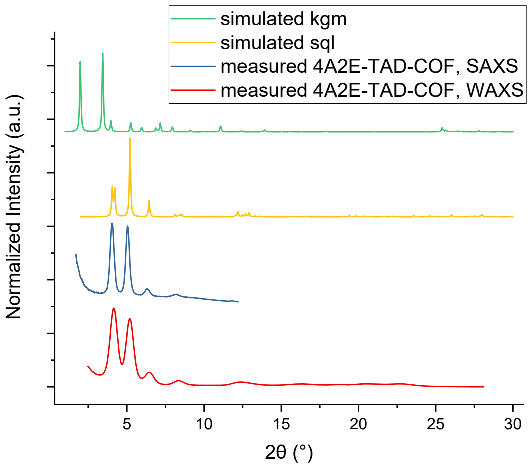

Figure S 15: PXRD measurement of the 4A2E-TAD-COF in small and wide angle configuration. Comparison with simulated *kgm* and *sql* PXRD spectra.

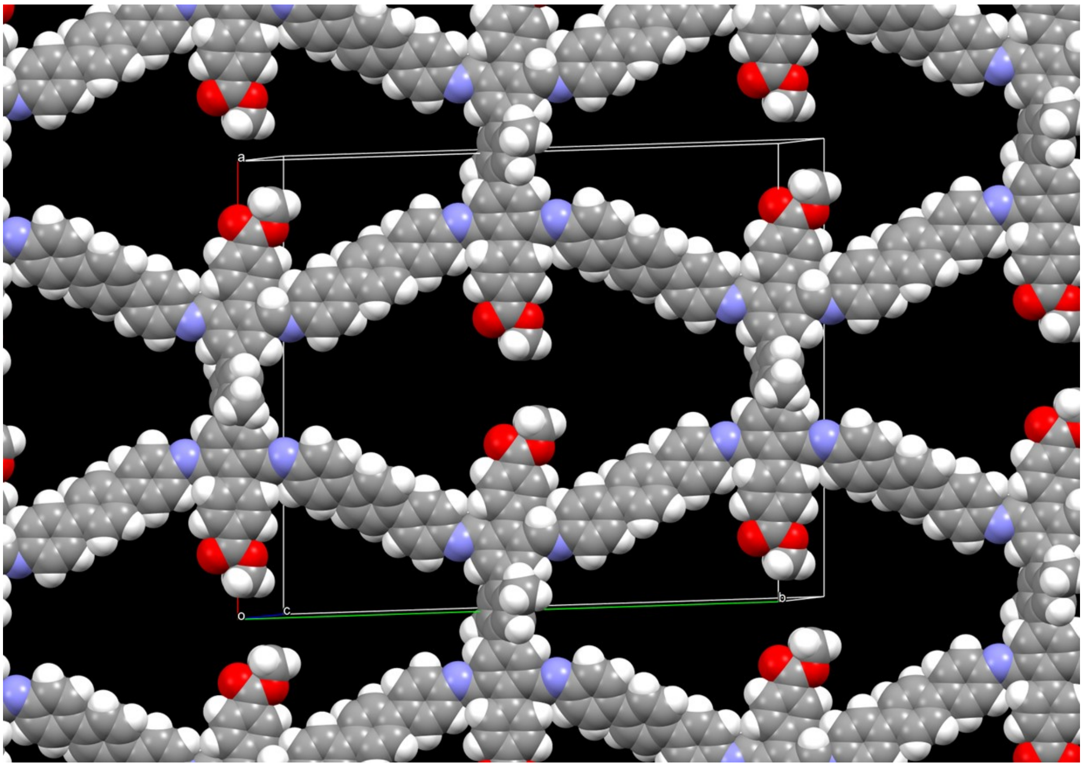

Figure S 14: Depiction of the 4,4''-Diamino-p-terphenyl based 4A2E-TAD-COF in the *sql* network.

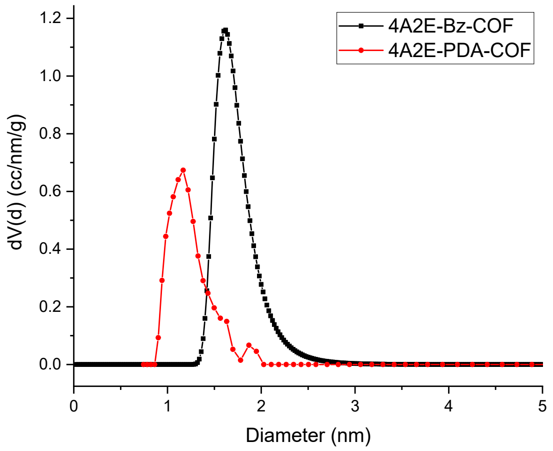

Figure S 16: Pore size distributions of the 4A2E-Bz-COF and the 4A2E-PDA-COF.

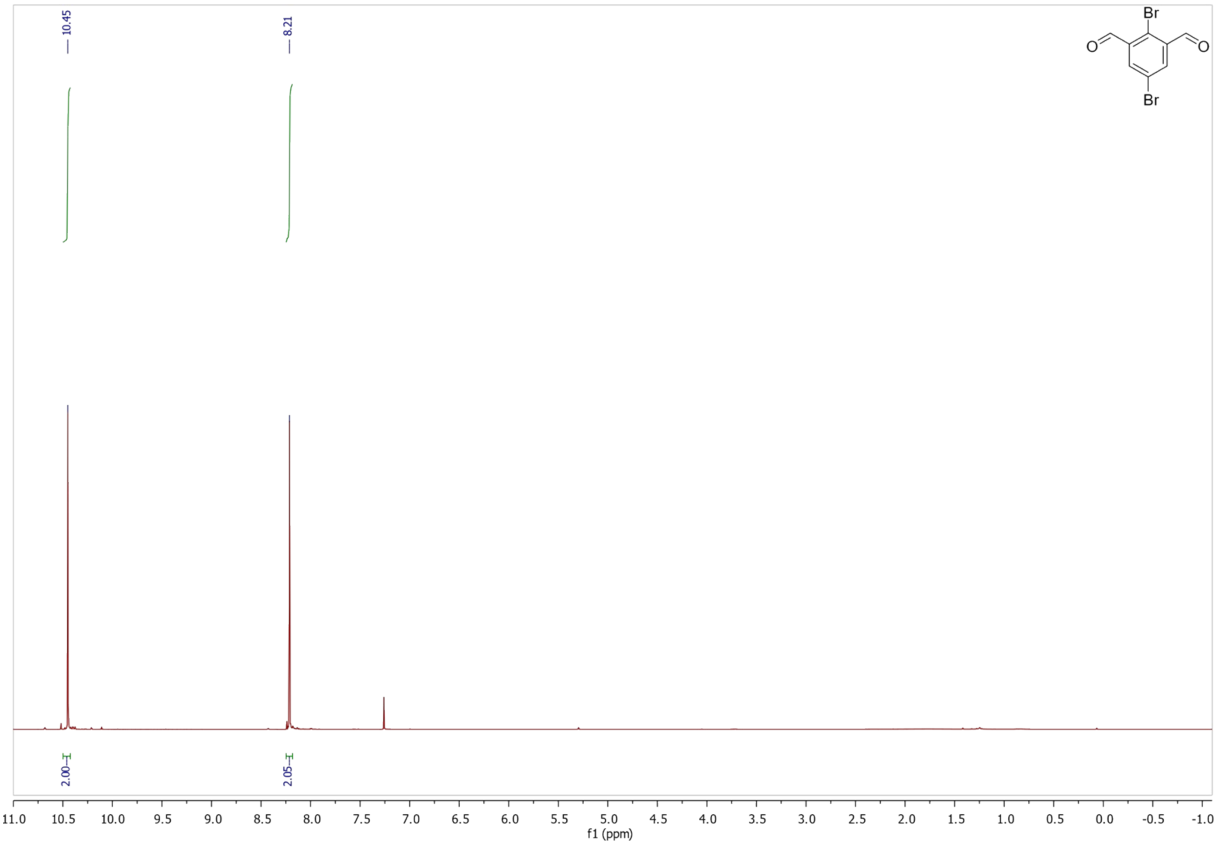

Figure S 17: ^{1}H NMR of 2,5-Dibromoisophthalaldehyde

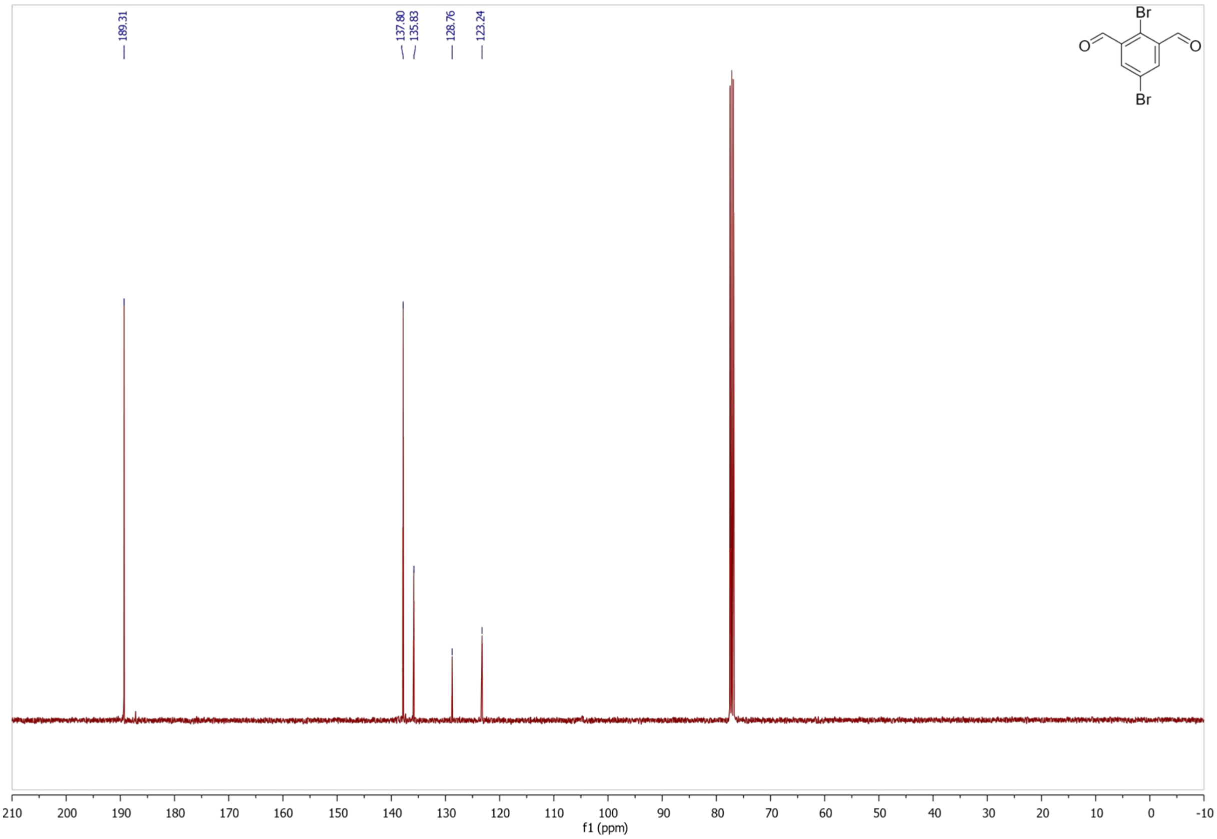

Figure S 18:^{13}C NMR of 2,5-Dibromoisophthalaldehyde

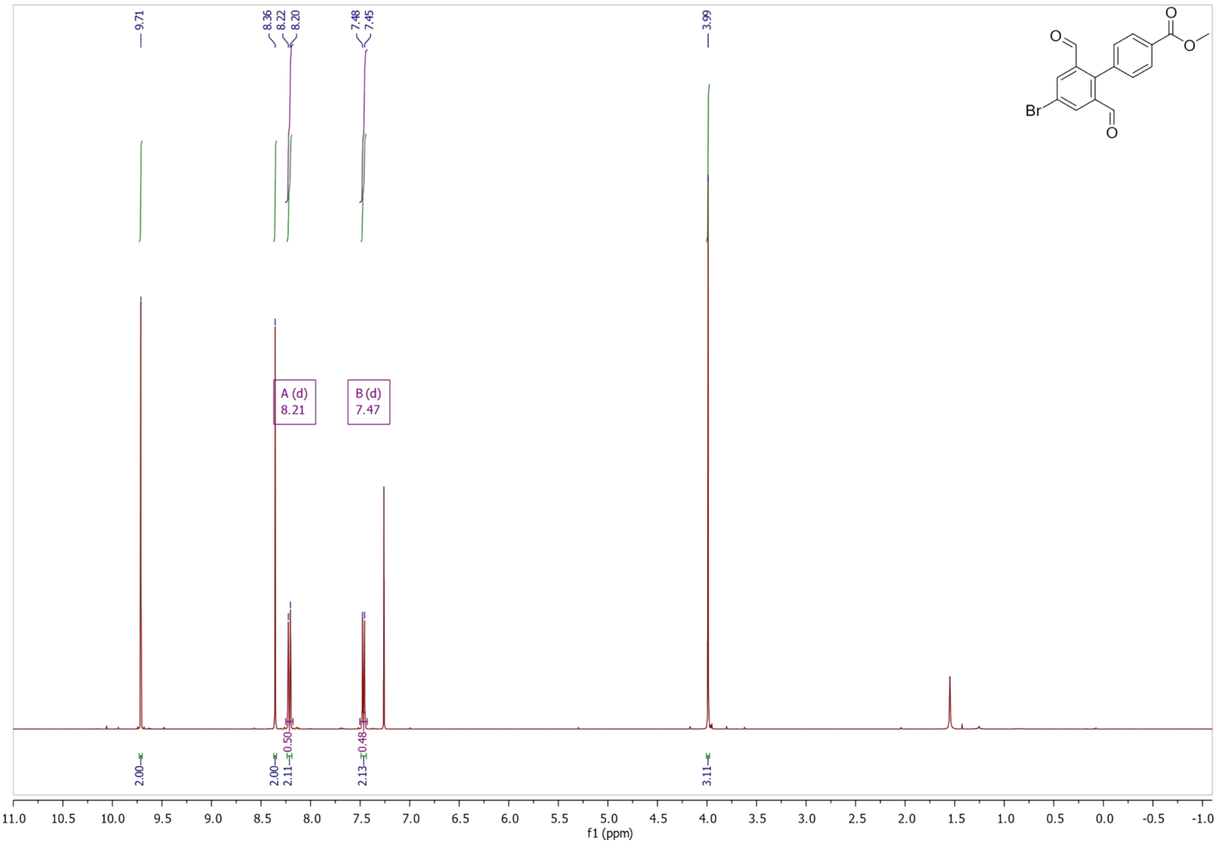

Figure S 19: ^{1}H NMR of Methyl 4'-bromo-2',6'-diformyl-[1,1'-biphenyl]-4-carboxylate

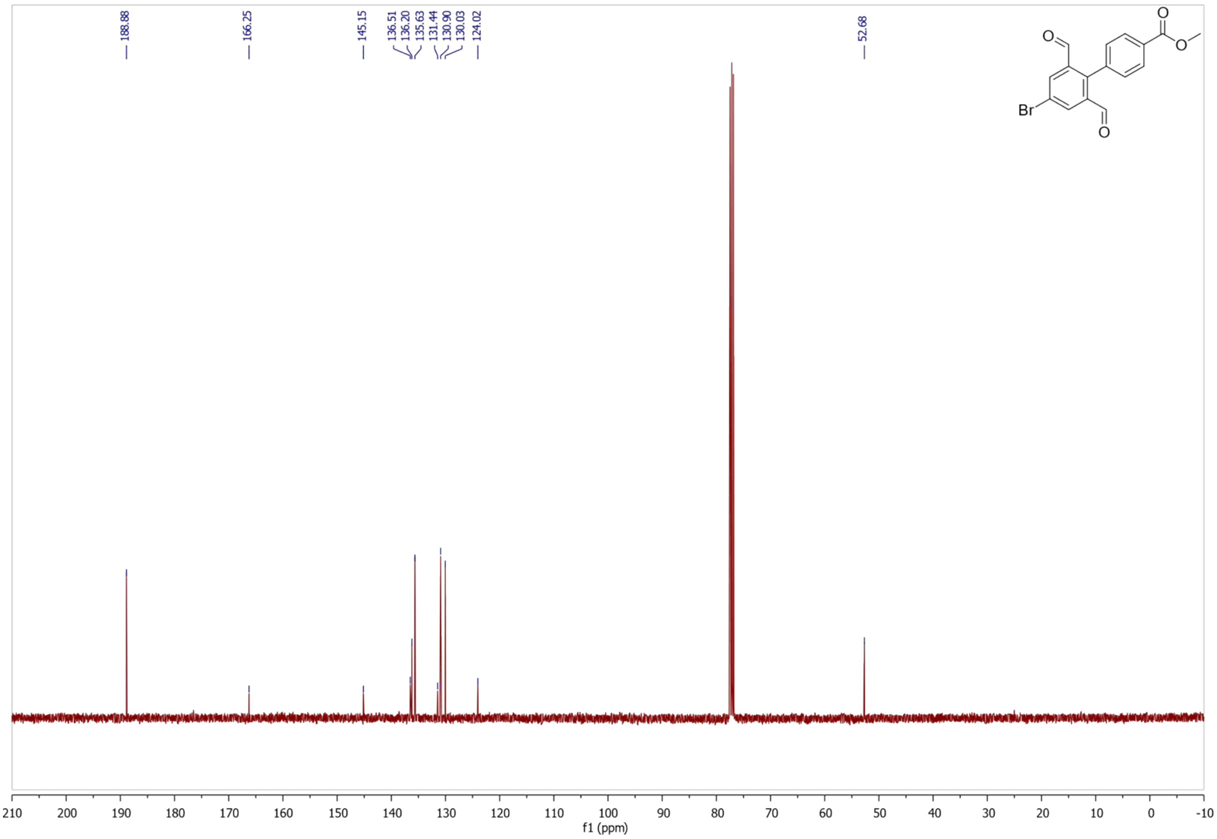

Figure S 20:^{13}C NMR of Methyl 4'-bromo-2',6'-diformyl-[1,1'-biphenyl]-4-carboxylate

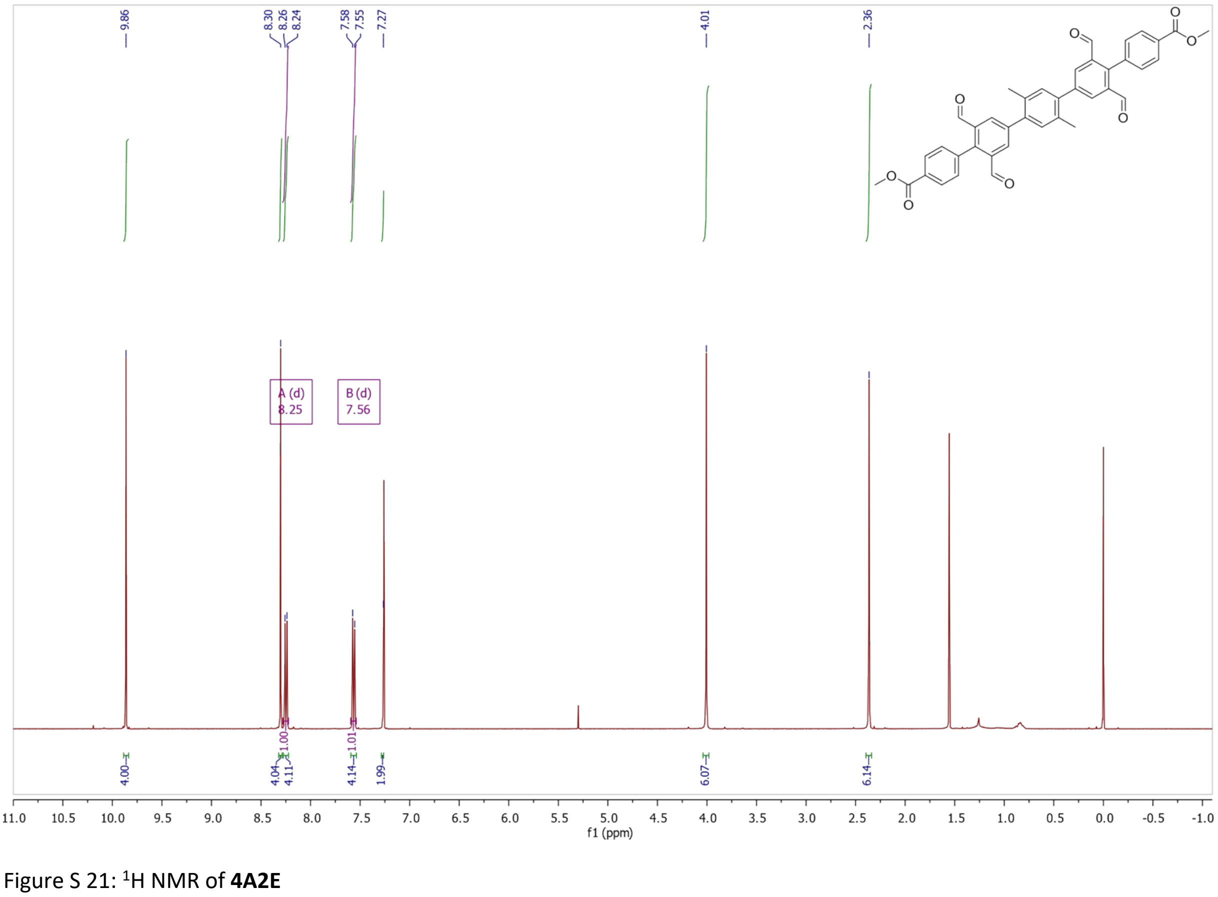

Figure S 21: ^{1}H NMR of **4A2E**

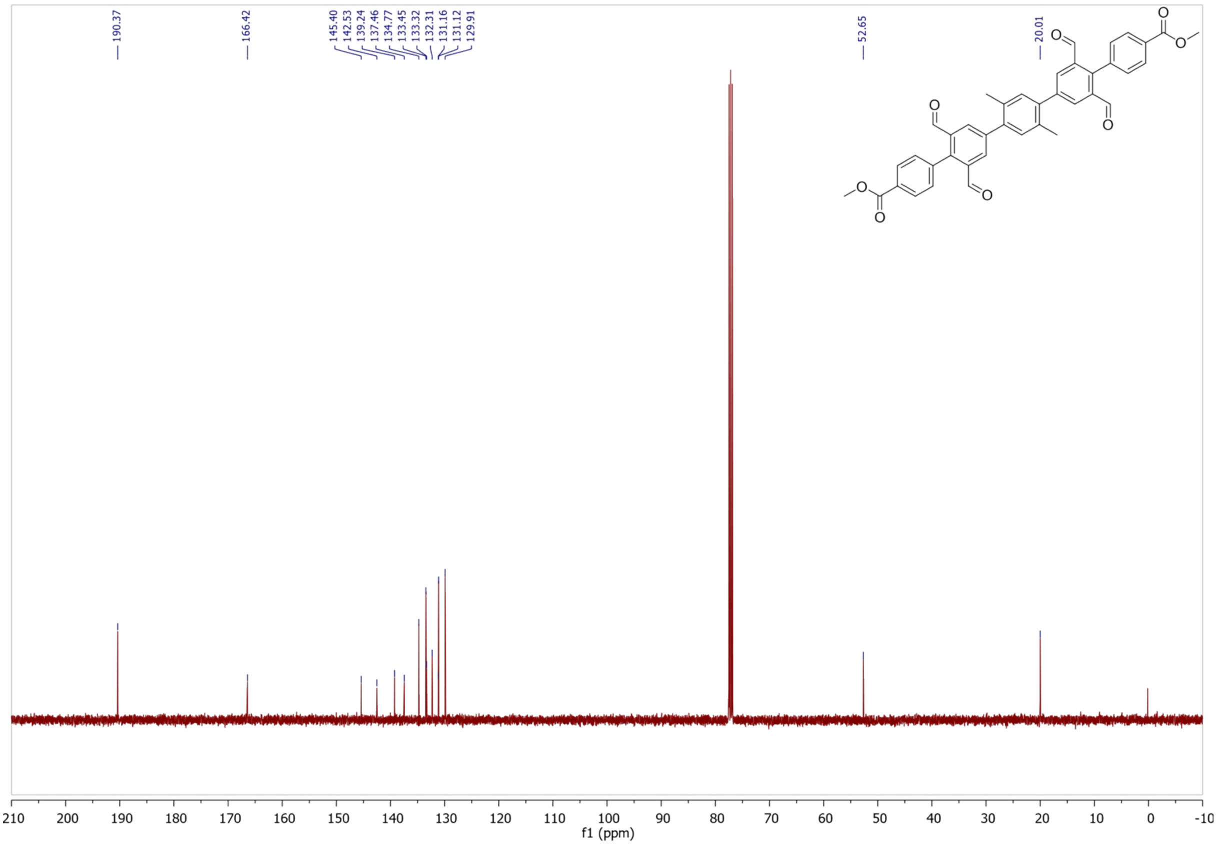

Figure S 22: ^{13}C NMR of **4A2E**

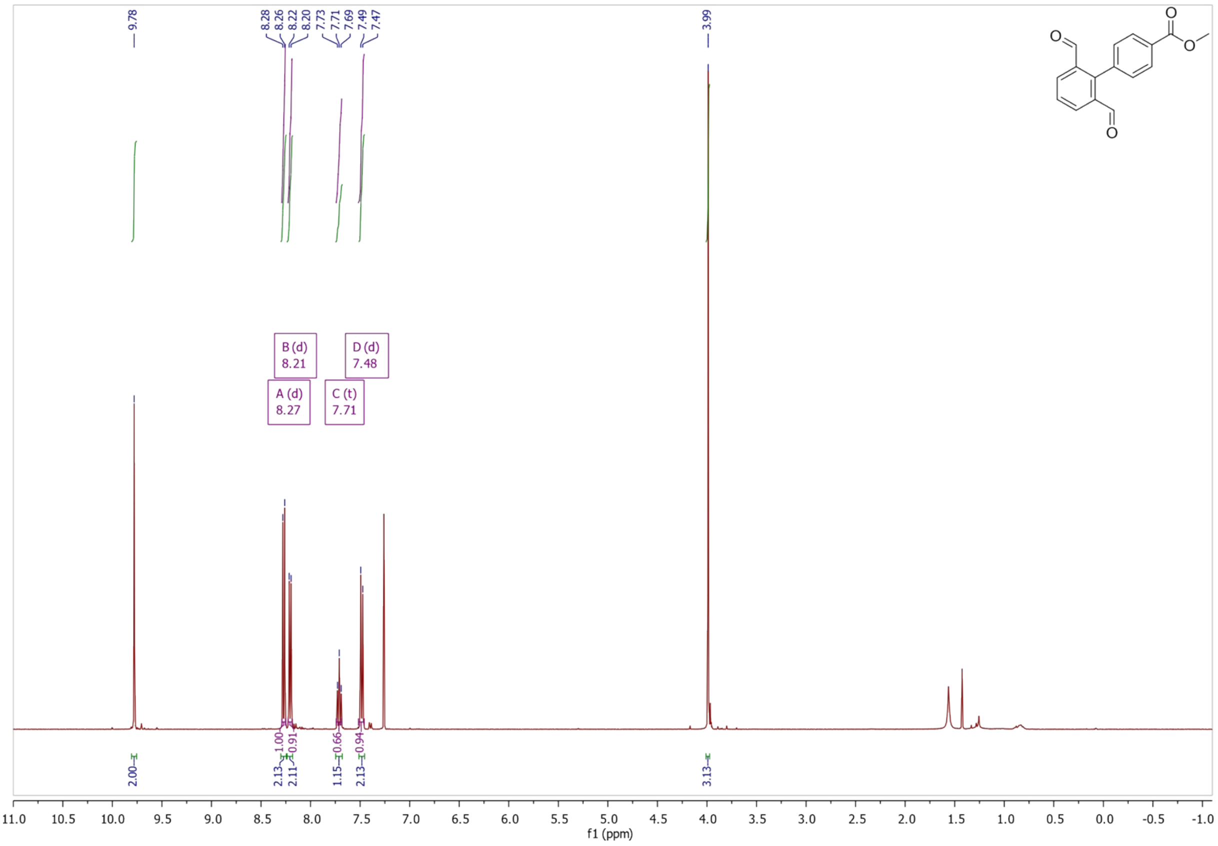

Figure S 23: ^{1}H NMR of Methyl 2',6'-diformyl-[1,1'-biphenyl]-4-carboxylate

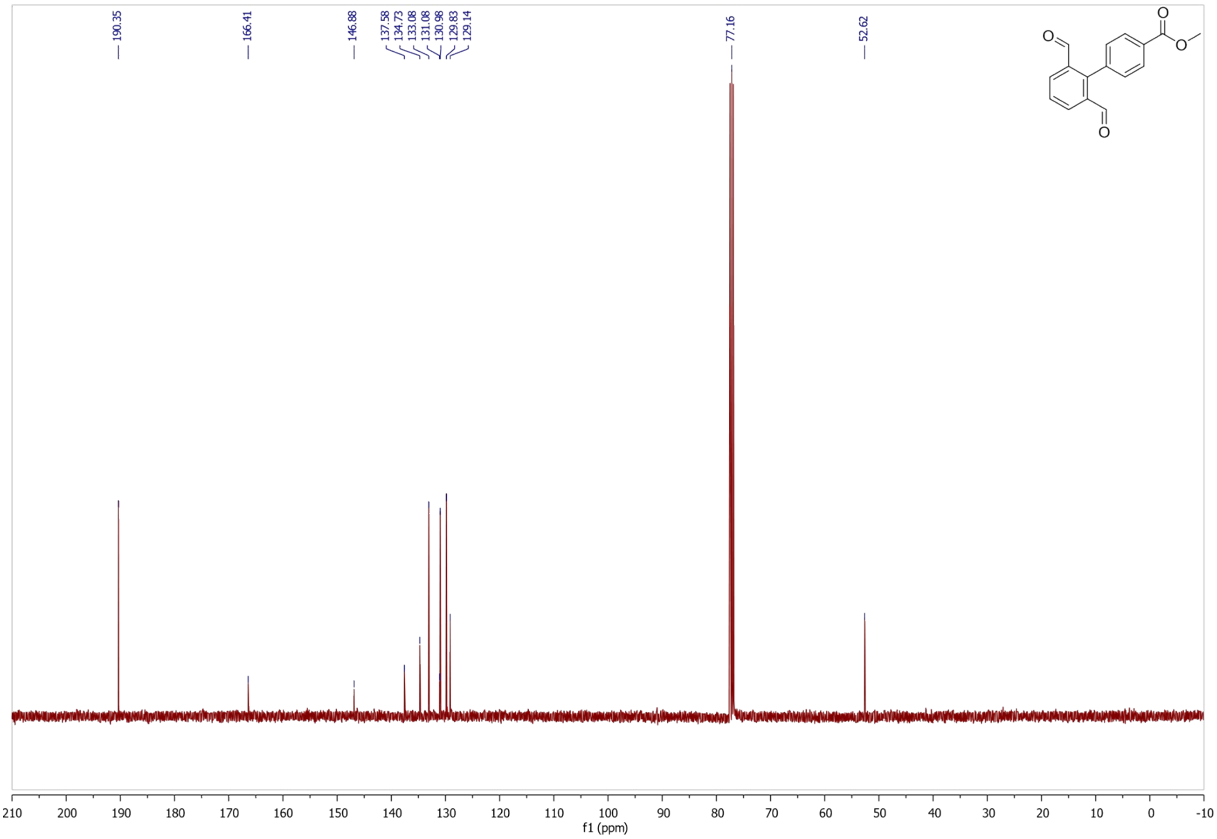

Figure S 24: ^{13}C NMR of Methyl 2',6'-diformyl-[1,1'-biphenyl]-4-carboxylate

Supplementary References

[1] J. A. Drewry, E. Duodu, A. Mazouchi, P. Spagnuolo, S. Burger, C. C. Gradinaru, P. Ayers, A. D. Schimmer, P. T. Gunning, *Inorg. Chem.* **2012**, *51*, 8284–8291.

[2] S. Grosjean, Z. Hassan, C. Wöll, S. Bräse, *Eur. J. Org. Chem.* **2019**, *2019*, 1446–1460.

[3] J. W. M. Osterrieth, J. Rampersad, D. Madden, N. Rampal, L. Skoric, B. Connolly, M. D. Allendorf, V. Stavila, J. L. Snider, R. Ameloot, J. Marreiros, C. Ania, D. Azevedo, E. Vilarrasa-Garcia, B. F. Santos, X.-H. Bu, Z. Chang, H. Bunzen, N. R. Champness, S. L. Griffin, B. Chen, R.-B. Lin, B. Coasne, S. Cohen, J. C. Moreton, Y. J. Colón, L. Chen, R. Clowes, F.-X. Coudert, Y. Cui, B. Hou, D. M. D'Alessandro, P. W. Doheny, M. Dincă, C. Sun, C. Doonan, M. T. Huxley, J. D. Evans, P. Falcaro, R. Ricco, O. Farha, K. B. Idrees, T. Islamoglu, P. Feng, H. Yang, R. S. Forgan, D. Bara, S. Furukawa, E. Sanchez, J. Gascon, S. Telalović, S. K. Ghosh, S. Mukherjee, M. R. Hill, M. M. Sadiq, P. Horcajada, P. Salcedo-Abraira, K. Kaneko, R. Kukobat, J. Kenvin, S. Keskin, S. Kitagawa, K. Otake, R. P. Lively, S. J. A. DeWitt, P. Llewellyn, B. V. Lotsch, S. T. Emmerling, A. M. Pütz, C. Martí-Gastaldo, N. M. Padial, J. García-Martínez, N. Linares, D. Maspoch, J. A. Suárez del Pino, P. Moghadam, R. Oktavian, R. E. Morris, P. S. Wheatley, J. Navarro, C. Petit, D. Danaci, M. J. Rosseinsky, A. P. Katsoulidis, M. Schröder, X. Han, S. Yang, C. Serre, G. Mouchaham, D. S. Sholl, R. Thyagarajan, D. Siderius, R. Q. Snurr, R. B. Goncalves, S. Telfer, S. J. Lee, V. P. Ting, J. L. Rowlandson, T. Uemura, T. Iiyuka, M. A. van der Veen, D. Rega, V. Van Speybroeck, S. M. J. Rogge, A. Lamaire, K. S. Walton, L. W. Bingel, S. Wuttke, J. Andreo, O. Yaghi, B. Zhang, C. T. Yavuz, T. S. Nguyen, F. Zamora, C. Montoro, H. Zhou, A. Kirchon, D. Fairen-Jimenez, *Adv. Mater.* **2022**, *34*, 2201502.

[4] O. V. Dolomanov, L. J. Bourhis, R. J. Gildea, J. a. K. Howard, H. Puschmann, *J. Appl. Crystallogr.* **2009**, *42*, 339–341.

[5] G. M. Sheldrick, *Acta Crystallogr. Sect. Found. Adv.* **2015**, *71*, 3–8.

[6] G. M. Sheldrick, *Acta Crystallogr. Sect. C Struct. Chem.* **2015**, *71*, 3–8.
